# Improvement of End-of-Synthesis Radiochemical Purity of ^177^Lu-DOTA-PSMA-Ligands with Alternative Synthesis Approaches: Conversion Upswing and Side-Products Minimization

**DOI:** 10.3390/pharmaceutics16121535

**Published:** 2024-11-30

**Authors:** Anton Larenkov, Iurii Mitrofanov, Marat Rakhimov

**Affiliations:** State Research Center—Burnasyan Federal Medical Biophysical Center of Federal Medical Biological Agency, Zhivopisnaya Str., Bld. 46, 123098 Moscow, Russia; mitrofanoff.yura@yandex.ru (I.M.); marat.rakhimov89@gmail.com (M.R.)

**Keywords:** lutetium-177, DOTA, PSMA, [^177^Lu]Lu-PSMA-617, radiochemical yield, radiochemical purity, related impurities, microwave heating, mixed media, ethanol

## Abstract

Background: Radiochemical purity is a key criterion for the quality of radiopharmaceuticals used in clinical practice. The joint improvement of analytical methods capable of identifying related radiochemical impurities and determining the actual radiochemical purity, as well as the improvement of synthesis methods to minimize the formation of possible radiochemical impurities, is integral to the implementation of high-tech nuclear medicine procedures. PSMA-targeted radionuclide therapy with lutetium-177 has emerged as an effective treatment option for prostate cancer, and [^177^Lu]Lu-PSMA-617 and [^177^Lu]Lu-PSMA^I&T^ have achieved global recognition as viable radiopharmaceuticals. Recently, it was shown that specific radiochemical impurities can form during the synthesis of [^177^Lu]Lu-PSMA-617 because of a spontaneous, thermally mediated condensation of the Glu-C(O)-Lys fragment, resulting in the formation of three different cyclic forms (with no affinity for PSMA). During this study, we identified another impurity, a product of detachment of the Glu-CO fragment from PSMA-617, caused by heating. The total content of all four thermally mediated degradation products may reach 9–11% during classical incubation for 30 min at 95 °C, reducing the radiochemical purity to an unacceptable level (albeit with high levels of radiochemical conversion). It is reasonable to assume that the formation of similar impurities is characteristic of all PSMA-specific vectors that contain Glu-C(O)-Lys pharmacophores. Because the formation of these impurities directly depends on the temperature and incubation time, to reduce their content in the reaction mixture at the end of the synthesis, it is necessary to select conditions to achieve a high level of radiochemical conversion for the minimum possible time and/or at the minimum sufficient temperature. Methods: In this study, using [^177^Lu]Lu-PSMA-617 as an example, we evaluated the efficiency of alternative methods of synthesis with microwave heating and co-solvent (ethanol) addition to ensure radiochemical yield and radiochemical purity in the shortest possible time and at the minimum necessary and sufficient synthesis temperature. Results: Both approaches achieved a significant reduction in the impurities content, while achieving satisfactory synthesis yields in a short time. In addition to improving the synthesis parameters and radiochemical purity, the use of microwave heating and the addition of ethanol reduces the negative influence of other auxiliaries on labeling kinetics. Notably, the addition of ethanol under certain conditions allowed [^177^Lu]Lu-PSMA-617 to be synthesized at room temperature for only 10 min. This makes it possible to achieve exceptionally high real radiochemical purity of the preparations, determined only by the quality of the original precursor. The approaches considered in this study can be successfully applied to improve the synthesis process and quality parameters of the finished product, both for known radiopharmaceuticals and for those under development.

## 1. Introduction

Advances in the technology and methodology of medical radionuclides and radiolabelled vector molecules for oncological treatment, together with the development of theranostic concepts, have allowed a clear justification of the potential of radiopharmaceutical therapy (RPT) against a variety of cancers in clinical practice [1,2,3]. RPT has further evolved towards precision medicine to enhance treatment response and has demonstrated efficacy in cancer treatment, particularly in cases in which conventional therapeutic strategies have proven ineffective [4,5]. This type of therapeutic care, which targets both primary malignancies and distant metastases, is increasingly being acknowledged as a highly effective, safe, and economically viable therapeutic approach that attracts renewed interest from both large pharmaceutical corporations and emerging biotech firms [6,7,8].

The main part of the nomenclature for therapeutic radiopharmaceuticals (both those under development and those already in clinical use) is represented by dosage forms that contain β^−^-emitting radionuclides. Despite the growing interest in new β^−^-emitting radionuclides (e.g., ^161^Tb) as well as α-emitters (such as ^225^Ac), lutetium-177 appears to be one of the most promising because of its nuclear properties, proven methods of obtaining the required quantities, and results of clinical use [8,9,10,11,12,13]. Two radiopharmaceuticals based on ^177^Lu have been widespread accepted and approved for use in clinical practice with marketing authorization: [^177^Lu]Lu-DOTA-TATE (oxodotreotide, Lutathera^®^ [14]; RPT of neuroendocrine tumors) and [^177^Lu]Lu-PSMA-617 (vipivotide tetraxetan, Pluvicto™ [15]; RPT for metastatic castrate-resistant prostate cancer). Research and development of new therapeutic radiopharmaceuticals with ^177^Lu are actively ongoing.

Prostate cancer (PCa) is one of the leading global health burdens owing to the increase in incidence, morbidity, mortality, and disability-adjusted life years in recent decades [16]. PCa remains the second most commonly diagnosed neoplasm among men worldwide [17]. PSMA-targeted radionuclide therapy with lutetium-177 has emerged as an effective treatment option for metastatic castration-resistant prostate cancer (mCRPC), even in patients with advanced disease [18,19]. Because most patients had repeated relapses before receiving PSMA-RPT, the results obtained so far are objectively reassuring [20,21]. [^177^Lu]Lu-PSMA-617 [15] and [^177^Lu]Lu-PSMA^I&T^ [22,23] have achieved global recognition as viable targeted treatments for prostate cancer [24]. Both [^177^Lu]Lu-PSMA-617 and [^177^Lu]Lu-PSMA^I&T^ RPT demonstrated favorable safety in patients with mCRPC (with comparable mean absorbed tumor doses) [25]. The impressive success of the clinical application of ^177^Lu-PSMA-ligands has motivated scientists to develop new molecules of this class to increase specificity and improve pharmacokinetic parameters to further improve patient care [26,27,28,29,30,31,32].

Radiochemical purity (RCP) is a key quality criterion for radiopharmaceuticals used in clinical practice. It is important to remember that the concept of radiochemical purity includes the entire range of possible and definable chemical forms of a radionuclide, not only the amount of radionuclide that has entered the complex formation reaction, which characterizes only the level of radiochemical yield (RCY). The sources and causes of the formation of radiochemical impurities that reduce the RCP of the finished dosage form can be different: initial radionuclide speciation and incomplete conversion to the desired chemical form; chemical changes during storage (including radiolysis processes); and chemical processes occurring under the influence of synthesis conditions (acidity, temperature, and concentration of auxiliary components). In the first case, which is more related to the radiochemical yield, radiochemical impurities differ significantly in nature from the target radioconjugate (unbound ionic or radiocolloidal forms of the radionuclide). It is the impurities formed as a result of chemical transformations of the target radioconjugate (or vector molecule) that constitute the greatest difficulty, both in analysis and in finding processes to suppress or avoid their formation.

In this context, a particularly interesting and illustrative example is the [^177^Lu]Lu-PSMA-617 molecule, which is the most studied and actively used worldwide. Whereas during storage of the finished dosage form, the main source of impurity formation is radiolysis (which can be suppressed by a suitable quencher) [33,34], during synthesis additional impurities are caused by thermally/chemically mediated changes in the vector molecule. Recently, Martin et al. showed that specific radiochemical impurities can be formed during the synthesis of [^177^Lu]Lu-PSMA-617 because of a spontaneous, thermally mediated condensation reaction of the Glu-C(O)-Lys fragment, resulting in the formation of three different cyclic forms under the dissociation of water [35]. The authors demonstrated the lack of affinity of the cyclized forms for the PSMA-receptor in in vitro experiments, and HPLC analysis of urine samples in studies on patients showed rapid excretion of these forms by the kidneys. The presence of these impurities reduces RCP and receptor-specific accumulation of radioactivity from the finished dosage form in the target foci.

It is reasonable to assume that the formation of similar impurities is characteristic of all PSMA-specific molecular vectors containing Glu-C(O)-Lys pharmacophore (as has been noted for [^18^F]F-PSMA-1007 [36], [^177^Lu]Lu-PSMA^I&T^ [37], and [^18^F]F-PSMA-617-NODA [38]).

In the framework of this study, using [^177^Lu]Lu-PSMA-617 as the most well-known example of a DOTA-conjugated PSMA-ligand with Glu-C(O)-Lys pharmacophore, we evaluated the efficiency of alternative methods of synthesis with microwave heating and co-solvent (ethanol) addition to ensure a high radiochemical yield and radiochemical purity in the shortest possible time and/or at the minimum necessary and sufficient synthesis temperature.

## 2. Materials and Methods

### 2.1. Chemicals and Reagents

Only 18.2 MΩ·cm deionized water (Milli-Q Millipore, Merck, Darmstadt, Germany) was used. All chemicals and solvents used in the synthesis were of ultrahigh purity or pharmaceutical grade.

Hydrochloric acid solution (30% Ultrapur; Supelco/Merck, St. Louis MO, USA), sodium acetate (≥99.999% trace metal basis, anhydrous; Honeywell, Seelze, Germany), and Tris base (≥99.9995% trace metal basis; Honeywell, Seelze, Germany) were used to prepare the reaction media with the required pH and buffer agent concentration. Lutetium(III) chloride (99.9%, anhydrous trace metal basis) was purchased from Acros Organics (Geel, Belgium). Absolute ethanol (USP, BP, Ph. Eur.) Pharma grade was purchased from Panreac Quimica (Barcelona, Spain). 2,3- and 2,5-dihydroxybenzoic acids (2,3-DHBA and 2,5-DHBA) were purchased from Sigma-Aldrich/Merck (St. Louis, MO, USA). Sep-Pak Accell Plus CM Plus Light Cartridges (130 mg sorbent per cartridge) were purchased from Waters Corporation (Milford, MA, USA).

The precursors PSMA-617 and PSMA^I&T^ were kindly provided by Jenguro Ltd. (Moscow, Russia) and the Center of Molecular Research (Moscow, Russia).

All organic solvents used for TLC/HPLC analysis were of HPLC gradient grade.

### 2.2. Lutetium-177

No-carrier-added lutetium-177 as a [^177^Lu]LuCl_3_ solutions in dilute hydrochloric acid were obtained from the following:

Radiopreparat State Enterprise (Tashkent, Republic of Uzbekistan) as a solution in 0.04 M HCl with an activity of 73.1 GBq/mL and a specific activity of 86.7 Ci/mg;

Research Institute of Atomic Reactors (Dimitrovgrad, Russia) as solutions in 0.04 M HCl with an activity of 286 GBq/mL and a specific activity of 92.9 Ci/mg.

### 2.3. Radiochemical Synthesis

In all cases, the volume of the reaction mixture was fixed at 1 mL, 2 mL, or 5 mL. The synthesis was carried out in Eppendorf Safe-Lock tubes (1.5, 2.0, or 5 mL) at temperatures of 24.5 (RT), 50, 75, 82, and 95 °C with Eppendorf ThermoMixer^®^ C (Hamburg, Germany).

For microwave heating, the reaction was performed with a CEM Discover 908005 Microwave Synthesis System in Biotage^®^ Microwave Reaction Vials (Uppsala, Sweden).

To an aliquot of an aqueous solution of PSMA-617 (20 µL, 1 mg/mL), a solution of CH_3_COONa_aq_ (2 mol/L) was added. (In a separate series of experiments, tris(hydroxymethyl)aminomethane (Tris, 2 mol/L) was used instead.) Additionally, water, a solution of hydrochloric acid (0.05 or 4 mol/L), an appropriate aliquot of ^nat^LuCl_3_ solution in 0.05 M HCl_aq._, and an aliquot of [^177^Lu]LuCl_3_ solution were added. The choice of the concentration of added hydrochloric acid (0.05 or 4 mol/L) depended on the chosen final concentration of the buffering agent at a fixed sample volume and desired pH. Ethanol was added to the reaction mixture for an additional series of experiments.

The amount of reaction mixture components was varied to ensure the following:

(1) the molar ratio [M(Lu)]:[L(PSMA)] was 1:10 with the lutetium-177 activity in the sample of 200–500 MBq/mL and a PSMA-617 concentration of 20 μg/mL; when modeling samples with clinical activity, the molar ratio M:L was maintained at 1:2 (with a fixed PSMA-617 concentration of 20 μg/mL ≈ 19.2 μmol/L, this corresponds to lutetium-177 activity concentration of ~7.4 GBq/mL);
(2) the concentrations of the buffering agent (sodium acetate) were 0.03, 0.1, 0.25, 0.5 or 1.0 mol/L;
(3) the pH of the final mixture was 4.6 ± 0.1 for all samples, except for the series of experiments studying the effect of acidity level on the radiochemical conversion kinetic (pH 6 and 8 in this series was maintained using Tris at the same concentration as sodium acetate);
(4) the volume fraction of ethanol was 0–80%.

In cases of synthesis with increased ^177^Lu activities (≥4.8 GBq/mL), stable lutetium was not added.

The activity was monitored with an ISOMED 2010 dose calibrator (MED Nuklear-Medizintechnik Dresden GmbH, Dresden, Germany).

### 2.4. TLC and HPLC Analysis of Preparations

Several radio-TLC methods were used to analyze the radiochemical conversion and radiochemical purity of [^177^Lu]Lu-PSMA preparations. The characteristics of the methods used are listed in Table 1.

Radiography of the TLC-strips was performed using a miniGita* radio-TLC scanner (Raytest, Straubenhardt, Germany) equipped with a beta detector based on a plastic scintillation probe as well as Cyclone^®^ Plus storage phosphor system (PerkinElmer, Waltham, MA, USA).

HPLC analysis of [^177^Lu]Lu-PSMA-617 preparations was carried out using the following:

LC-20AD Prominence chromatograph (Shimadzu Co. Ltd., Kyoto, Japan) equipped with diode array detector SPD-M20A and MiniScanPRO TLC/HPLC Flow-Count system (Eckert & Ziegler Eurotope GmbH, Berlin, Germany) with FC-3200 NaI/PMT and FC-3600 Plastic Scintillator/PMT based detectors (∅ 0.25 mm × 5 cm flow cell, PEEK tubing).

LicArt-62 chromatograph (Labconcept LLC, Saint Petersburg, Russia), equipped with a diode array detector and flow radioactivity detector GABI Nova basic (2 × 2” NaI-PMT detector, Elysia-Raytest, Straubenhardt, Germany) with a 5 µL measuring cell.

Two methods were used to analyze the radiochemical purity of the [^177^Lu]Lu-PSMA preparations in this study (Table 2). The reversed phase C_18_ columns were purchased from Phenomenex^®^ (Torrance, CA, USA). In both methods, solvent A was 0.1% (*v*/*v*) TFA in water (TFA—trifluoroacetic acid, HPLC grade, Panreac Quimica, Barcelona, Spain) and solvent B was 0.1% (*v*/*v*) TFA in acetonitrile (HPLC gradient grade, Fisher Scientific, Loughborough, UK). The columns were thermostated at 30 °C.

### 2.5. Mass Spectrometry for Identification of Side Products

A Waters ACQUITY Premier UPLC system equipped with a diode array detector and Waters ACQUITY HSS T3 column (100 × ⌀2.1 mm, 1.8 µm) was used for chromatographic separation of the components. Chromatography was carried out in gradient mode 0-1-16 min = 1-1-40% B (0.3 mL/min, A—0.042% formic acid in water, B—0.050% formic acid in acetonitrile), followed by UV detection at 205 and 268 nm and mass spectrometry using high-resolution tandem quadrupole time-of-flight mass spectrometry with an ion-mobility analyzer (Waters SYNAPT XS HDMS, Waters, Milford, MA) with positive ionization in the electrospray and MS^E^ data acquisition mode. The range of measurements was 20–2000 *m*/*z*, and the energy of the colliding particles in the collision cell was 4 eV in the low-energy regime and varied in the range of 20–45 eV in the high-energy regime. The *m*/*z* values of positively charged ions are given in sum with the mass of escaping electrons, which is considered when performing mass spectrometer calibration. The system was calibrated with leucine/enkephalin (0.1 µg/mL, *m*/*z* 556.2771).

## 3. Results

### 3.1. HPLC Analysis of ^177^Lu-PSMA Preparations and Identification of Side-Products

**Figure 1:**
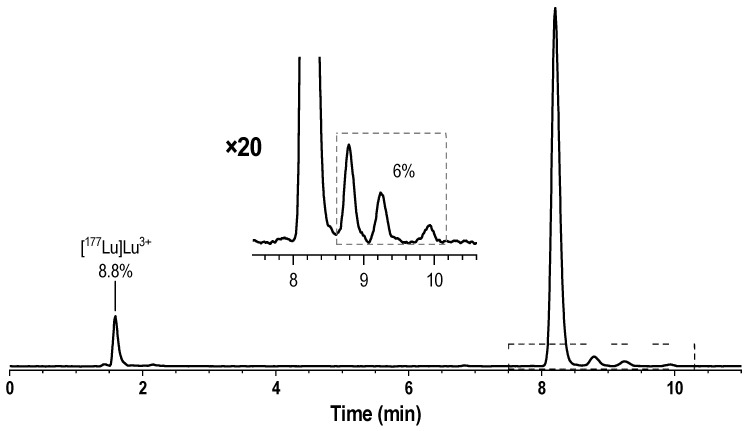
Radio-HPLC chromatograms of the model sample [^177^Lu]Lu-PSMA-617 synthesized at 95 °C for 15 min (with the addition of 10% free [^177^Lu]Lu^III^), obtained with Method 1. RCP with correction to free lutetim-177 is 94%.

Initially, to determine the presence of thermocyclization impurities in ^177^Lu-PSMA preparations, we used two HPLC methods (see Section 2): Method 1 was similar to the method proposed by Martin et al. [35], but using Phenomenex Luna 250 × ⌀4 mm (100 Å) instead of Phenomenex Jupiter Proteo^®^ 250 × ⌀4.6 mm (90 Å); Method 2, as modification of the method, we proposed earlier using a Phenomenex Luna 150 × 4 mm (100 Å) [34,39]. A typical result of the analysis of a model [^177^Lu]Lu-PSMA-617 preparation obtained using method 2 is shown in Figure 1.

Overall, the results of the analysis were consistent with expectations, and both methods clearly identified three impurity peaks after the main peak of [^177^Lu]Lu-PSMA-617. However, despite the fact that Method 2 used a shorter column, the developed gradient program allowed for better separation of impurities and [^177^Lu]Lu-PSMA-617 (the peak/valley ratio was increased by more than eight times); therefore, in further experiments, Method 2 was chosen as the main one (see Supplementary Materials Figure S1).

**Figure 2:**
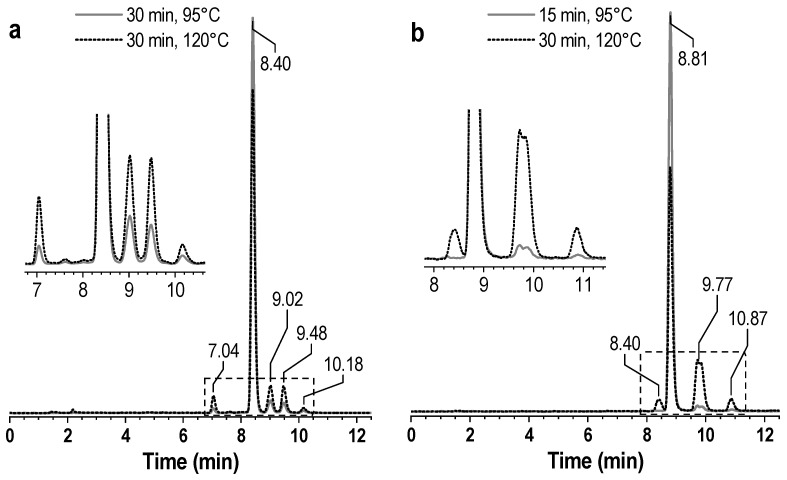
Radio-HPLC chromatograms of the [^177^Lu]Lu-PSMA-617 (**a**) and [^177^Lu]Lu-PSMA^I&T^ (**b**) samples obtained at different synthesis temperatures.

An analysis of a large number of ^177^Lu-PSMA preparations obtained under various conditions showed that, in addition to the three known impurities, another impurity with lower lipophilicity (*R_t_* = 7 min), which was not previously described, was also identified in the chromatograms. The content of this impurity also depended on incubation time and temperature. In the case of [^177^Lu]Lu-PSMA-617, the content of this impurity became noticeable (approaching 1–2% with a total content of thermally mediated degradation products of 9–11%) during classical incubation for 30 min at 95 °C. After autoclaving the reaction mixture at 120 °C, the amount of this impurity increased to 4–5%, and the total content of the thermally mediated degradation products was 19–21%—Figure 2a. A similar picture was observed for the synthesis of [^177^Lu]Lu-PSMA^I&T^, with the only difference being that the resolution parameters of the observed impurities were changed in the implemented analysis method—Figure 2b.

**Figure 3:**
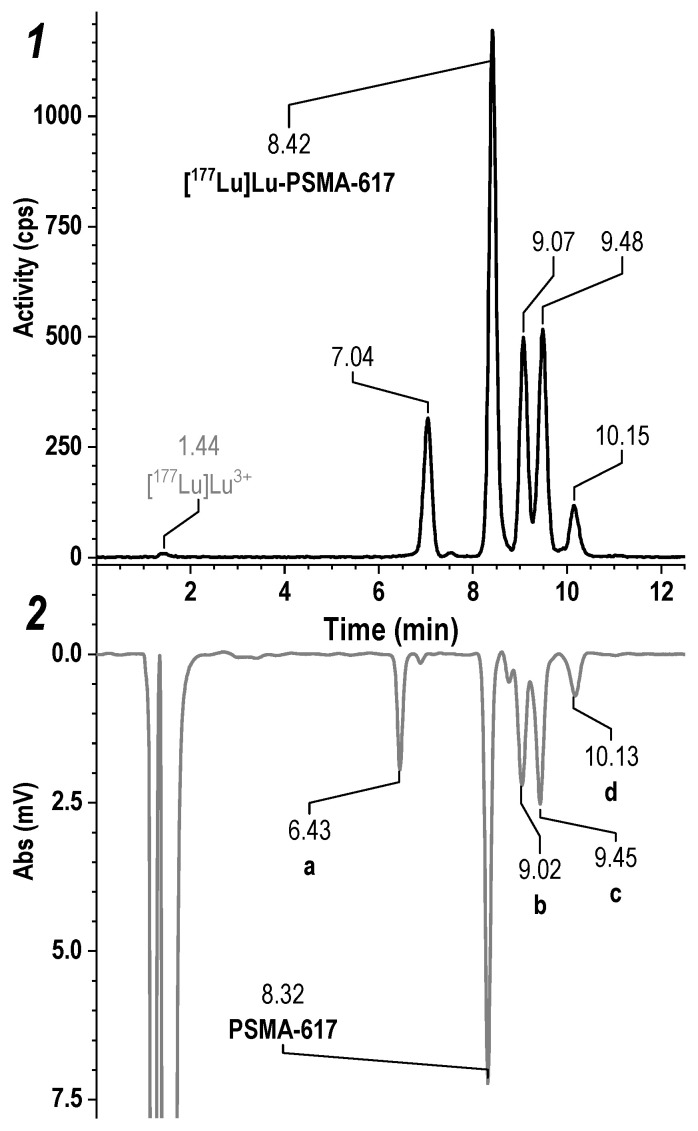
Results of HPLC analysis of the [^177^Lu]Lu-PSMA-617 sample obtained by prolonged intensive heating for 2 h at 120 °C in Method 2 with radiometric (**1**) and UV (**2**) detection (220 nm with baseline correction by blank analysis). The volume of the reaction mixture was 1 mL, the amount of PSMA-617 was 100 μg, the activity of ^177^Lu was 150 MBq, and the solution contained 0.03 M sodium acetate at pH 4.5.

To identify this unknown impurity, a series of [^177^Lu]Lu-PSMA-617 samples were synthesized by prolonged intensive heating (to obtain sufficient quantities of all impurities for qualitative analysis)—Figure 3.

**Figure 4:**
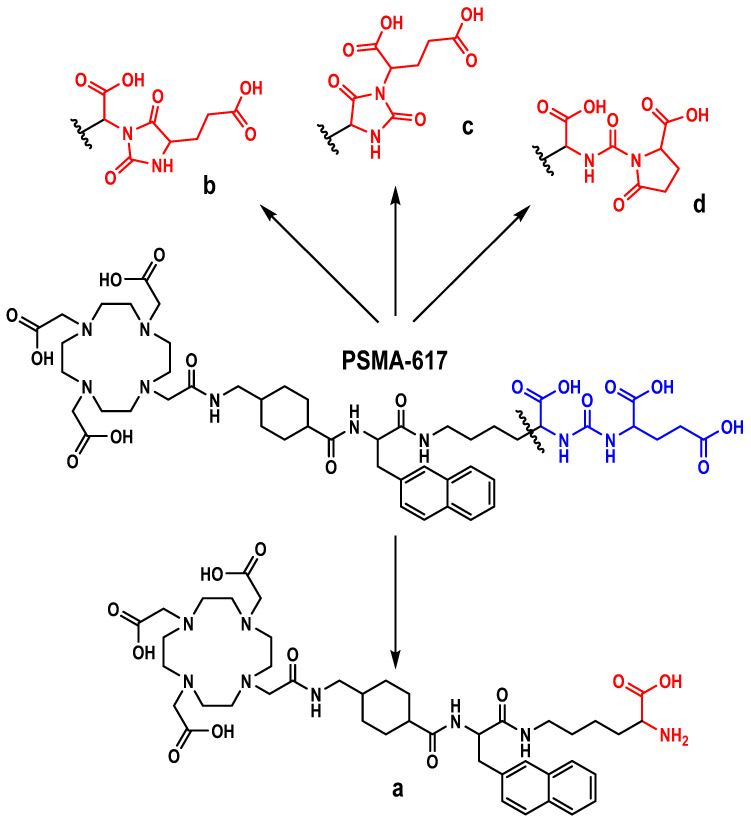
Structural formulae of PSMA-617 and its thermal-mediated degradation products: **b**, **c**, **d**—derivatives of hydantoin ‘A’, hydantoin ‘B’, and pyroglutamic acid, respectively; **a**—cleavage product of the Glu-CO fragment.

The samples were analyzed using high-resolution tandem quadrupole time-of-flight mass spectrometry with an ion-mobility analyzer (Waters SYNAPT XS HDMS). In addition to three known products of thermocyclization (molecules with pyroglutamic acid fragment and two hydantoin derivatives instead of Glu-C(O)-Lys pharmacophore—Figure 3, peaks b-c-d), a new impurity, a product of detachment of the Glu-CO fragment from PSMA-617, was also reliably identified—Figure 3, peak a (see Supplementary Materials Figures S2–S9). It is fair to assume that this impurity, due to the degradation of the pharmacophore, will not have specificity for the target receptor as well as thermocyclisation products. Thus, its content should also be controlled, and the total content of thermal degradation impurities should be minimized during the implemented synthesis approaches. The structures of all identified impurities are shown in Figure 4.

Since the formation of all the above impurities directly depends on the temperature and incubation time of the reaction mixture, in order to reduce their content in the reaction mixture at the end of the synthesis, it is necessary to select conditions to achieve a high level of radiochemical conversion for the minimum possible time and/or at the minimum sufficient temperature.

### 3.2. Radiochemical Conversion and Kinetics of Lutetium-177 Incorporation into the Structure of PSMA-617

**Figure 5:**
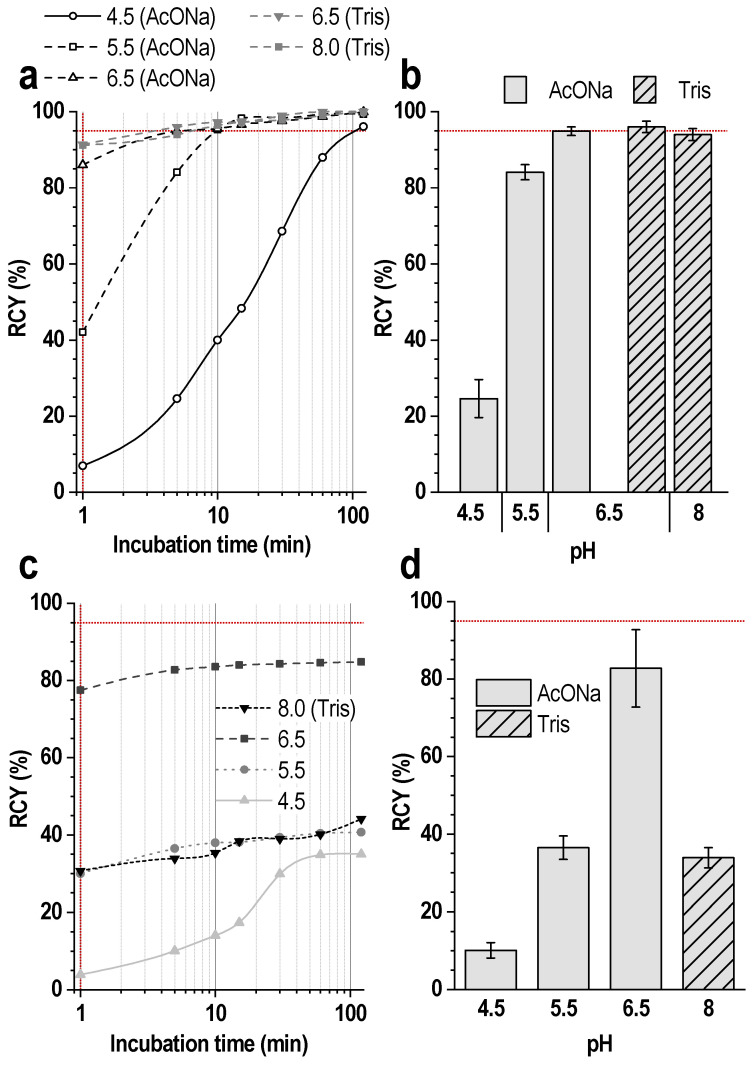
(**a**) Kinetics of [^177^Lu]Lu-PSMA-617 formation at different pH values (error bars are omitted for clarity); (**b**) The magnitude of the radiochemical yield after 5 min of incubation of the reaction mixture at different pH values. RCY values are presented as the mean ± SD, *n* = 3. Synthesis conditions for (**a**,**b**) volume—1 mL, buffer (AcONa/Tris) concentration—0.03 mol/L, 200 MBq/mL of ^177^Lu, 20 μg of PSMA-617, ^nat^LuCl_3_ added, [Lu]:[PSMA] = 1:10, ambient (24.5 °C) temperature. (**c**) Kinetics of [^177^Lu]Lu-PSMA-617 formation at different pH values (error bars are omitted for clarity); (**d**) The magnitude of the radiochemical yield after 5 min of incubation of the reaction mixture at different pH values. RCY values are presented as the mean ± SD, *n* = 3. Synthesis conditions for (**c**,**d**): volume—1 mL, buffer (AcONa/Tris) concentration—0.25 mol/L, 500 MBq/mL of ^177^Lu, 20 μg of PSMA-617, ^nat^LuCl_3_ added, [Lu]:[PSMA] = 1:2, C[Lu] = 9.6 μmol/L (equivalent~7.4 GBq/mL of ^177^Lu), ambient (24.5 °C) temperature.

In radiolabeling with lutetium-177, for most ligands based on DOTA-conjugated molecules, the pH of the reaction mixture is generally accepted to be 4.0–4.5. At the same time, with increasing pH, the degree of deprotonation of the chelating agent (DOTA) increases, thereby increasing the kinetics of complex formation. The experimental data obtained for dilute solutions with low concentrations of ^177^Lu are consistent with this and show the possibility of achieving high radiochemical yields with increasing pH already at ambient temperature—Figure 5a,b.

However, when scaling up the synthesis and increasing the concentration of lutetium-177 activity, carrying out the reaction at elevated pH (>6.5) is complicated by the hydrolysis and precipitation of the radionuclide—Figure 5c,d.

In turn, increasing the temperature to improve the radiochemical yield may also lead to an intensification of the hydrolysis processes and a decrease in the initial pH value of the colloid precipitation (see Section 4). Hence, despite the attractiveness of the reaction kinetics at elevated pH, further experiments were performed at pH 4.5 ± 0.1 in an acetate buffer medium.

**Figure 6:**
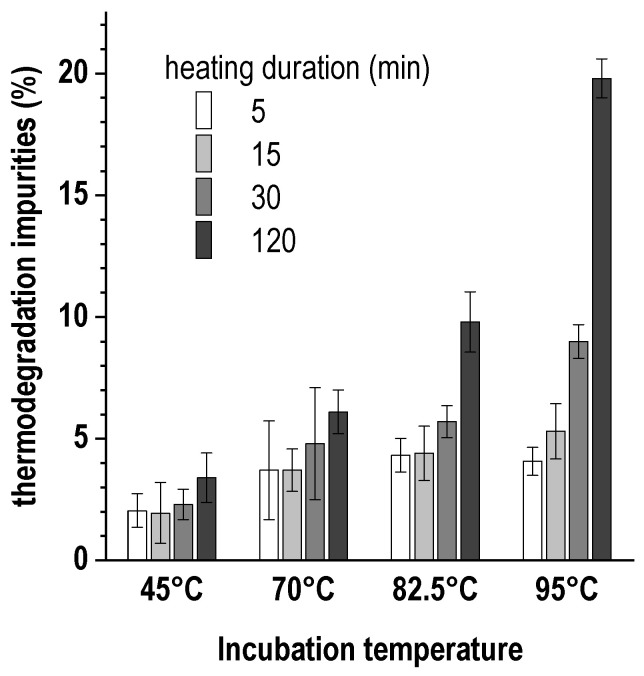
Effect of incubation duration and heating intensity of the reaction mixture (1 mL, 20 μg of PSMA-617, 0.03 mol/L sodium acetate, 200 MBq of ^177^Lu; pH 4.5 ± 0.1) on accumulation of [^177^Lu]Lu-PSMA-617 thermodegradation impurities. The data were corrected for radiochemical conversion values (mean ± SD, *n* = 5).

Figure 6 presents the data on the rate of accumulation of the aforementioned thermodegradation impurities as a function of the duration and intensity of heating of the reaction mixture.

**Figure 7:**
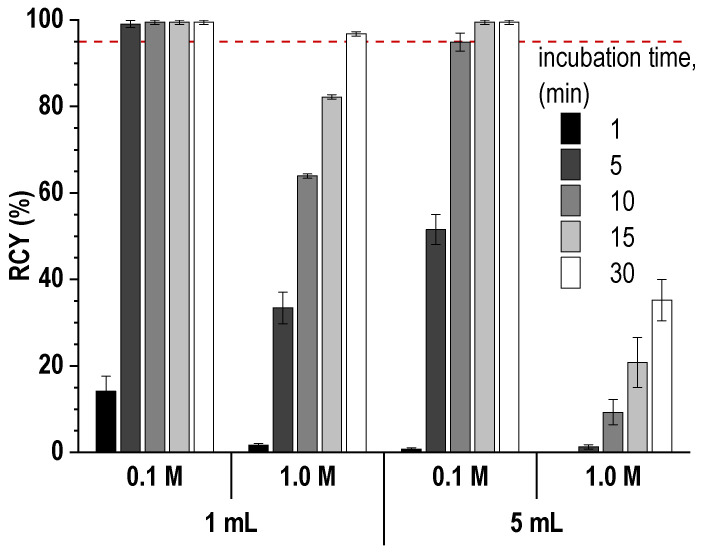
Effect of reaction mixture volume on the rate of lutetium-177 incorporation into the PSMA-617 structure at buffer concentrations of 0.1–1.0 M (sodium acetate, pH 4.5 ± 0.1) and 70 °C (20 μg of PSMA-617, 200 MBq/mL of ^177^Lu, ^nat^LuCl_3_ added, [Lu]:[PSMA] = 1:10). RCY values are presented as the mean ± SD, *n* = 3.

**Figure 8:**
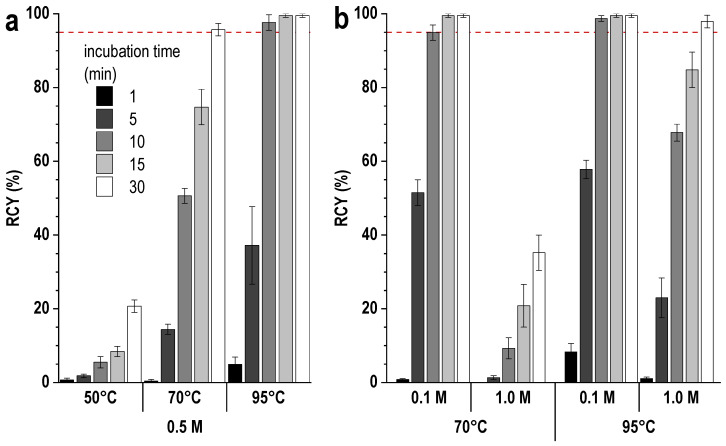
Effect of temperature (**a**) and buffering agent concentration (**b**) on the rate of lutetium-177 incorporation into the structure of PSMA-617. The volume of the reaction mixture was 5 mL, the amount of PSMA-617 was 20 μg, and the activity of ^177^Lu was 1000 MBq (molar ratio of [Lu]:[PSMA] = 1:10). RCY values are presented as the mean ± SD, *n* = 3.

Studies have shown that the impurity content can be reduced to 2.0 ± 0.5% by heating the reaction mixture to 45 °C for 15 min. Under these conditions and at a low concentration of the buffer agent, the level of radiochemical conversion exceeded 98%, and the total radiochemical purity was ≥96%. However, again, when scaling the synthesis procedure to the clinical activities of lutetium-177, such low buffer concentrations and a small volume of the reaction mixture may not be sufficient. Experimental data show that an increase in the concentration of the buffering agent, as well as an increase in the volume of the reaction mixture, requires an increase in either the incubation time or the temperature to achieve an acceptable level of radiochemical conversion—Figure 7 and Figure 8.

In turn, an increase in the reaction temperature is limited by the formation of radiochemical impurities caused by thermal degradation, and an increase in the synthesis time is limited by the absorbed dose formed in the volume of the reaction mixture and the formation of radiochemical impurities caused by radiolysis. In turn, the introduction of stabilizers-quenchers into the reaction mixture to suppress radiolysis (such as, for example, gentisic acid) also affects the kinetics of [^177^Lu]Lu-PSMA-617 complex formation—in the absence of heating, the reaction yield is only ~50% with the addition of gentisic acid—and requires an increase in the synthesis temperature (see Supplementary Materials Figure S10).

Hence, for reproducible improvement of [^177^Lu]Lu-PSMA-617 synthesis in terms of radiochemical yield and radiochemical purity, alternative synthesis methods can be used under the limitations of the possible formation of the indicated impurities.

### 3.3. Microwave-Supported Synthesis

**Figure 9:**
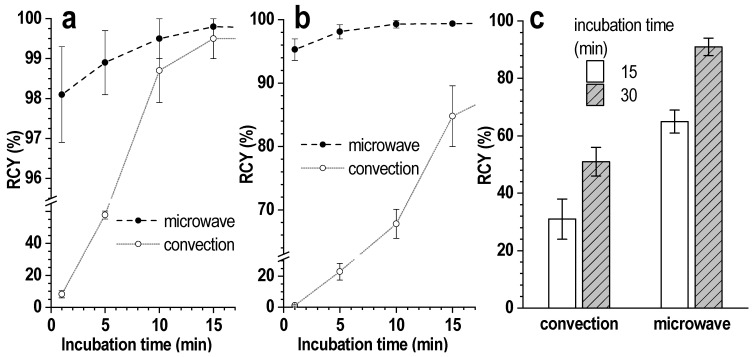
Kinetics of [^177^Lu]Lu-PSMA-617 formation under microwave and convection heating (95 °C) in 0.1 mol/L (**a**) and 1.0 mol/L (**b**) sodium acetate media (pH 4.5). The volume of the reaction mixture was 5 mL, the amount of PSMA-617 was 20 μg, the activity of ^177^Lu was 1000 MBq, ^nat^LuCl_3_ was added (molar ratio [Lu]:[PSMA] = 1:10); (**c**) RCY of [^177^Lu]Lu-PSMA-617 under microwave and convection heating (95 °C) with the addition of 2.5 μmol 2,3-DHBA. The volume of the reaction mixture was 1 mL, the amount of PSMA-617 was 50 μg, the activity of ^177^Lu was 700 MBq, 0.03 M sodium acetate (pH 4.5). RCY values are presented as the mean ± SD, *n* = 5.

The use of microwave heating for the synthesis of [^177^Lu]Lu-PSMA-617 resulted in a significant reduction in the time required for a satisfactory radiochemical yield, even in the case of a large volume of the reaction mixture and high concentration of auxiliary substances (i.e., buffer agent). Thus, with a reaction mixture volume of 5 mL and a precursor amount of 20 μg, radiochemical yields of ~98% were achieved in 1 (98.1 ± 1.2%) and 5 (98.2 ± 1.1%) min at sodium acetate concentrations of 0.1 and 1.0 mol/L, respectively—Figure 9a,b. For comparison, under the same conditions but in the case of conventional (convection) heating, at least 10 and 30 min are required at sodium acetate concentrations of 1.0 and 1.0 mol/L, respectively.

Microwave heating allows the achievement of high levels of radiochemical conversion, even under conditions where the target reaction is hampered by interfering substances. In addition to the concentration of the acetate buffer, this effect was clearly observed in the case of synthesis with the addition of 2,3-DHBA; in this case, the use of microwave heating guaranteed a nearly 2-fold increase in the radiochemical yield compared to convection heating—Figure 9c.

**Figure 10:**
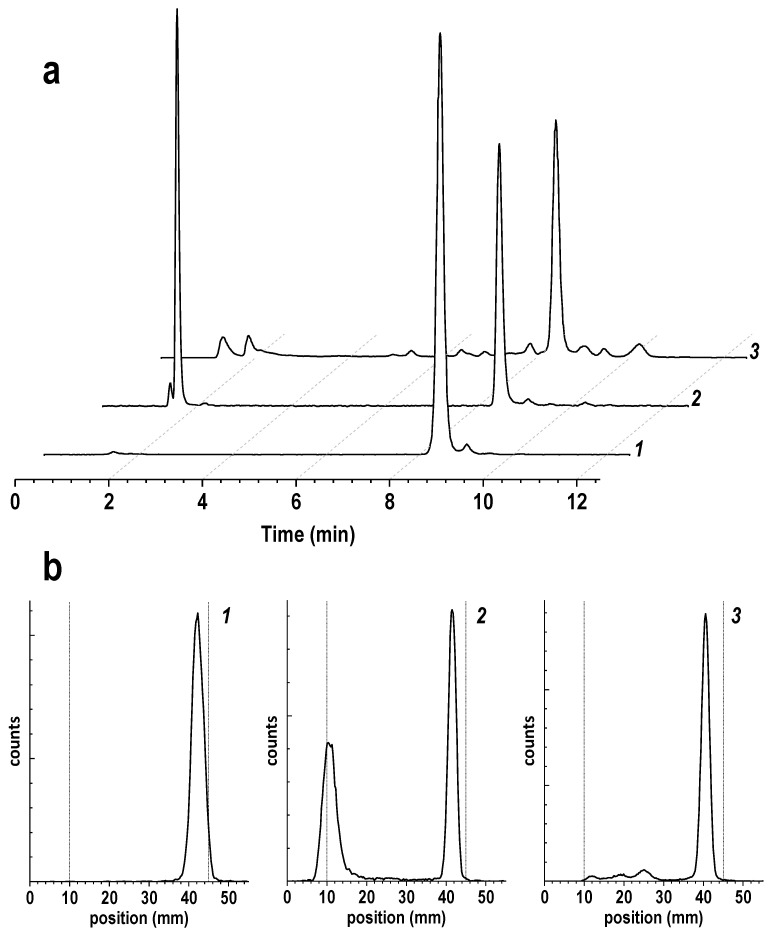
(**a**): Radio-HPLC chromatograms (method 2) of the [^177^Lu]Lu-PSMA-617 preparations obtained after 1 min of microwave heating at 95 °C (1), 1 min of convective heating at 95 °C (2), and 30 min of convective heating at 95 °C (3). (**b**): Corresponding Radio-TLC chromatograms (method 1). The volume of the reaction mixture was 1 mL, the amount of PSMA-617 was 100 μg, the activity of ^177^Lu was 4.8 GBq, 0.03 M sodium acetate (pH 4.5), and no quenchers were added.

Notably, the intensification of the formation rate of the target [^177^Lu]Lu-PSMA-617 complex observed during microwave heating did not lead to a proportional increase in the accumulation of thermocyclization and thermodegradation impurities. In addition, achieving almost quantitative radiochemical conversion in a short period of time minimizes the amount of radiochemical impurities caused by the radiolysis processes. Thus, carrying out the synthesis with lutetium-177 activities close to those required for clinical use under microwave heating for only one minute and without adding any quenchers gave a radiochemical yield of ≥99.5% and a radiochemical purity of ≥97,5% (2% of thermocyclization impurities were determined in the reaction mixture)—Figure 10. Conducting the synthesis using convection heating with a similar composition of the reaction mixture gave RCY (RCP) values of 50% (48%) and ≥99.5% (52%) after incubation for 1 and 30 min, respectively.

### 3.4. Synthesis in Ethanol-Containing Medium

**Figure 11:**
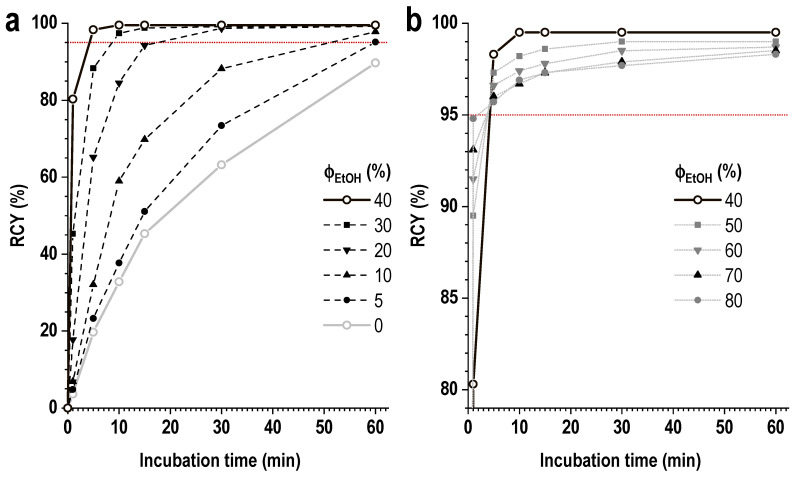
The effect of ethanol fraction in the reaction mixture on the rate of lutetium-177 incorporation into the PSMA-617 structure ((**a**) 0–40 vol.% of ethanol; (**b**) 40–80 vol.% of ethanol). The volume of the reaction mixture was 1 mL, the amount of PSMA-617 was 20 μg, the activity of ^177^Lu was 250 MBq, 0.03 M sodium acetate (pH 4.5), ^nat^LuCl_3_ added, [Lu]:[PSMA] = 1:10. The RCY values are presented as the mean for n = 3 (error bars are omitted for clarity).

The addition of ethanol to the reaction mixture significantly improved the kinetics of lutetium-177 incorporation into the PSMA-617 structure, with a proportional increase in the reaction yield with increasing ethanol fraction—Figure 11. Therefore, at the ethanol content of 40 vol.%, a nearly quantitative reaction yield (99.5 ± 0.5%) may be observed after 10 min of incubation even at ambient temperature (RT, 24.5 °C), compared to 32.8 ± 3.5%, in the case of an ethanol-free mixture (for 1 mL volume of the reaction mixture, 0.03 mol/L sodium acetate, pH 4.5, [Lu]:[PSMA] = 1:10).

Notably, further increase in the ethanol fraction to >40 vol.% allows, under given conditions, to achieve a reaction yield of ≥90% after just one minute. However, as the reaction rate increased, a slight decrease in its completeness was observed: if after one minute the values of the reaction yield at room temperature were 80.5 ± 2.1% (40 vol.% of ethanol) and 94.8 ± 0.5% (80 vol.% of ethanol), then after 10 min they were already 99.5 ± 0.5% (40 vol.%) and 96.9 ± 1.1% (80 vol.%), and after 30 min—99.8 ± 0.2% (40 vol.%) and 97.7 ± 1.5% (80 vol.%). Thus, achieving a complete yield of complex formation of lutetium-177 and PSMA-617 at ethanol concentrations greater than 40 vol.% is somewhat complicated (under the given conditions), and in the synthesis time interval of 5–15 min, a conditional maximum dependence of the radiochemical yield on the ethanol fraction was observed with 40 vol.% of ethanol (Figure S11).

**Figure 12:**
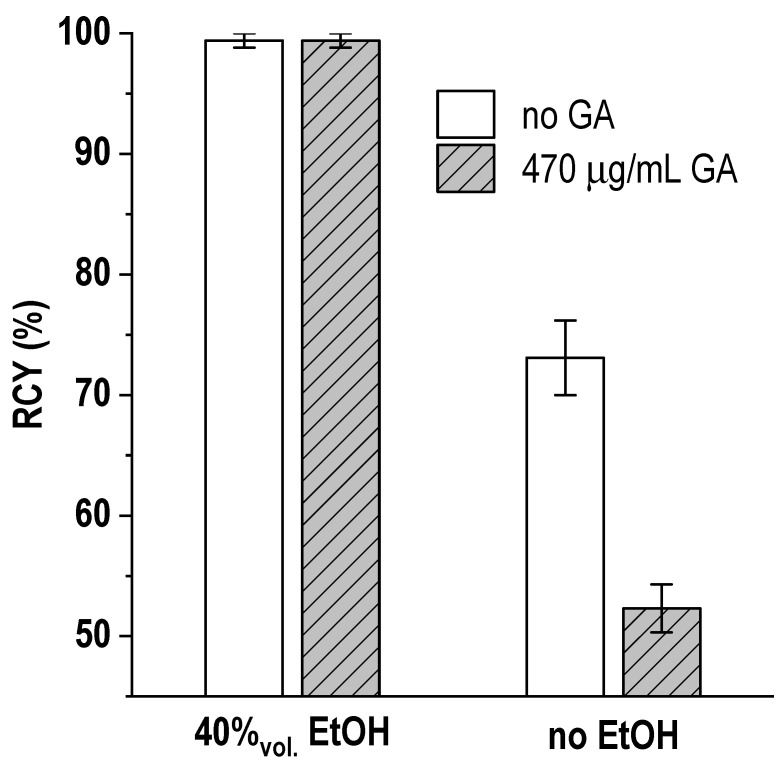
Dependence of the radiochemical yield of [^177^Lu]Lu-PSMA-617 synthesis at ambient temperature on the ethanol and gentisic acid (GA) content in the reaction mixture. The volume of the reaction mixture was 1 mL, the amount of PSMA-617 was 20 μg, the activity of ^177^Lu was 250 MBq, 0.03 M sodium acetate (pH 4.5), ^nat^LuCl_3_ added, [Lu]:[PSMA] = 1:10, 45 min incubation at 24.5 °C. RCY values are presented as the mean ± SD, *n* = 3.

Notably, in addition to the intensification of [^177^Lu]Lu-PSMA-617 complex formation, the presence of ethanol makes it possible to remove the negative effect of gentisic acid on the kinetics of the target reaction—Figure 12.

**Figure 13:**
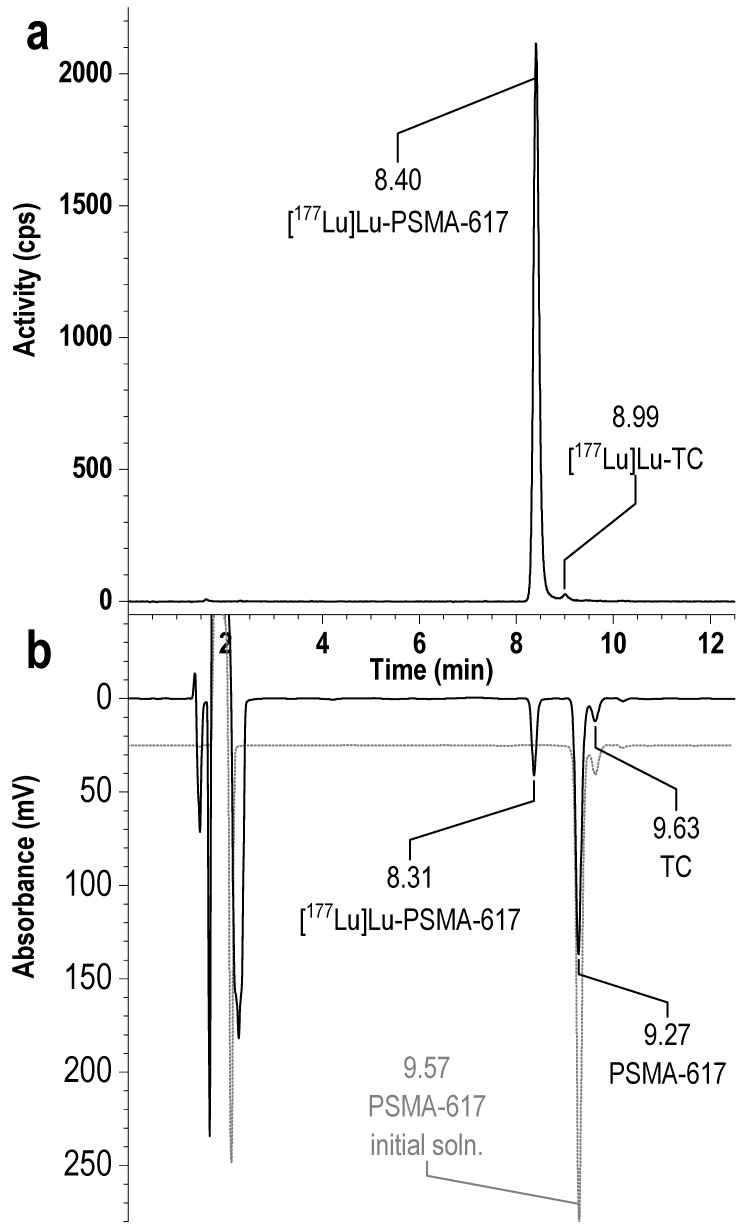
The results of HPLC analysis (Method 2, **a**—radioactivity, **b**—UV) of the [^177^Lu]Lu-PSMA-617 sample obtained with the addition of 40 vol.% ethanol after 15 min of incubation at ambient (24.5 °C) temperature. The volume of the reaction mixture was 1 mL, the amount of PSMA-617 was 20 μg, the activity of ^177^Lu was 250 MBq/mL, ^nat^LuCl_3_ added, [Lu]:[PSMA] = 1:5, 0.03 M sodium acetate, pH 4.5; 2% of thermocyclization (TC) was determined from the radiochromatogram of the preparation.

Hence, in the case of diluted acetate buffer (0.03 mol/L, pH 4.5), 40 vol.% ethanol additive allows the synthesis of [^177^Lu]Lu-PSMA-617 at room temperature, providing nearly quantitative radiochemical conversion (≥99.5%) and a real radiochemical purity of more than 97% within 10–15 min of incubation (Figure 13).

It is important to note that 2% of the thermocyclization impurities determined in the radio/UV-chromatograms of the obtained sample are obviously present in it initially, characterizing the quality of the precursor and not the implemented synthesis method.

It is fair to note that performing the synthesis in ethanol-containing media excludes the possibility of applying the final purification stage using the SPE C_18_-cartridge. However, this stage can be successfully replaced by the Sep-Pak Accell Plus CM cation exchange cartridge (for reliable removal of all unreacted lutetium-177, if present [40,41]). The experimental data showed that, with ethanol contents of 10 and 20 vol.%, an increase in the distribution coefficient (*D_g_*) of lutetium-177 on the Accell Plus CM sorbent to 458 ± 23 mg/mL and 430 ± 41 mg/mL, respectively, is observed (compared to 346 ± 17 mg/mL for the ethanol-free medium)—see Supplementary Materials Figure S12. Although a further increase in the ethanol content led to a sharp drop in the *D_g_* of lutetium-177 (30 vol.%—192 ± 41 mg/mL, 40 vol.%—22 ± 4 mg/mL), it is clear that in the case of synthesis, for example, at 40 vol.% ethanol content, only a 2-fold dilution of the reaction mixture is sufficient for the effective use of the CM cartridge as a final purification stage.

**Figure 14:**
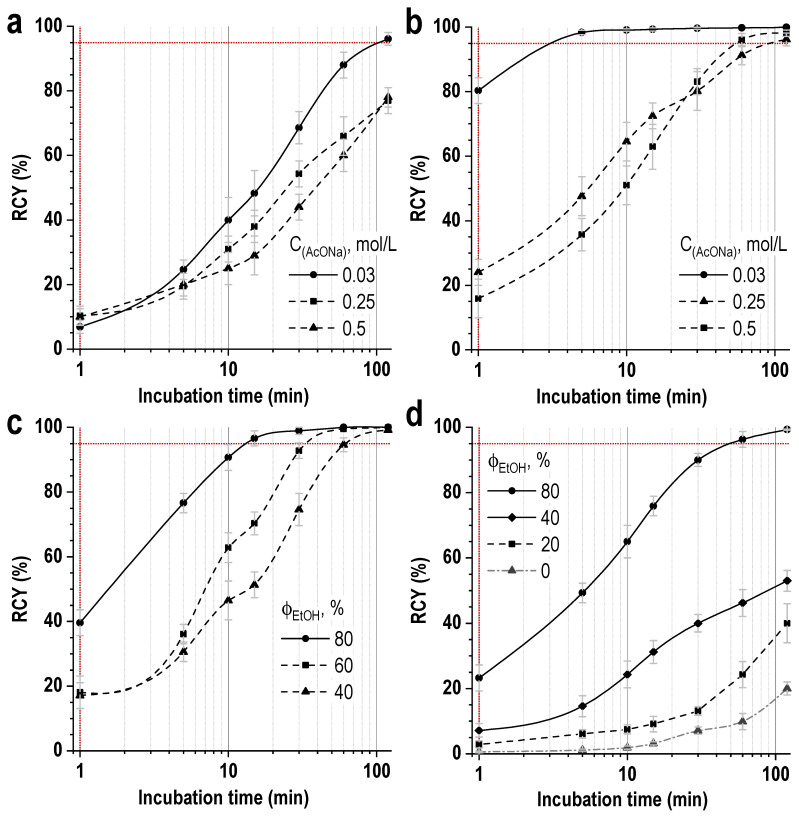
Kinetics of [^177^Lu]Lu-PSMA-617 formation under different conditions: (**a**) ethanol-free media, different concentrations of sodium acetate, pH 4.5, [Lu]:[PSMA] = 1:10; (**b**) 40 vol.% of ethanol, different concentrations of sodium acetate, pH 4.5, [Lu]:[PSMA] = 1:10; (**c**) different ethanol fractions, 0.25 mol/L sodium acetate, pH 4.5, [Lu]:[PSMA] = 1:10; (**d**) different ethanol fractions, 0.25 mol/L sodium acetate, pH 4.5, [Lu]:[PSMA] = 1:2; the volume of the reaction mixture was 1 mL, and the mixture was incubated at ambient temperature (24.5 °C).

It should be clearly noted that 40% ethanol is the conditional optimum (corresponding to a dilute acetate buffer at a given metal-to-ligand ratio). As indicated earlier (Figure 7 and Figure 8), increasing the concentration of the buffering agent affected the kinetics of [^177^Lu]Lu-PSMA-617 complex formation. In the case of an ethanol-containing medium, this dependence also remained, and an increase in the sodium acetate concentration required an increase in the ethanol content (more than 40 vol.%) to achieve comparable radiochemical yields at room temperature (Figure 14a–c). The same applies to an increase in the metal-to-ligand molar ratio (Figure 14d).

**Figure 15:**
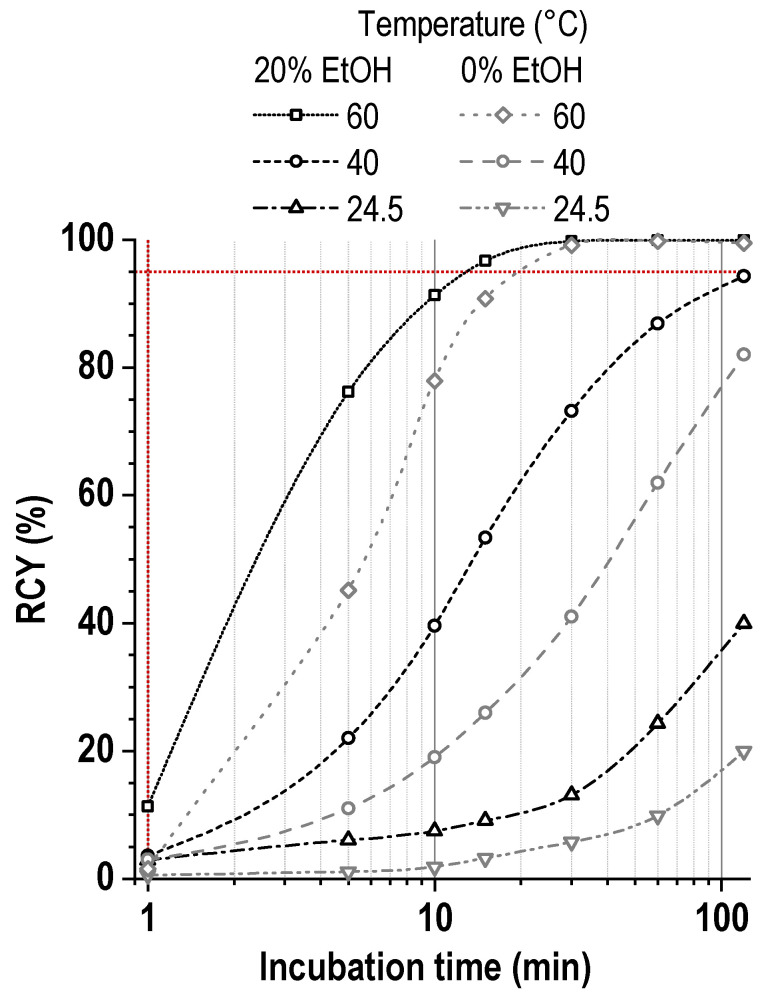
The kinetics of [^177^Lu]Lu-PSMA-617 formation (20 vol.% of ethanol, 0.25 mol/L sodium acetate, pH 4.5) at different temperatures in comparison with ethanol-free media. The reaction mixture volume was 1 mL, the activity of ^177^Lu was 250 MBq, ^nat^LuCl_3_ was added, and [Lu]:[PSMA] = 1:2. The RCY values are presented as the mean for *n* = 3 (error bars are omitted for clarity).

Thus, depending on the buffer concentration used and when it is undesirable to use increased amounts of ethanol in the reaction mixture, slight heating may still be necessary to achieve a satisfactory radiochemical yield in a short time (especially at a high metal-to-ligand molar ratio)—Figure 15. In this case, variation in metal-to-ligand ratio is another option to improve labeling kinetics (Supplementary Materials Figure S13).

## 4. Discussion

The preparation of clinically useful radiopharmaceutical samples with high radiochemical yields and a truly high level of radiochemical purity depends on both approaches implemented for synthesis and analysis. It is obvious that the use of fast, simple, and non-selective radio-HPLC methods (in the absence of established recommendations and pharmacopoeial requirements for impurity profiling) will not allow the determination of related impurities of ^177^Lu-DOTA-PSMA thermocyclization-thermodegradation described by Martin et al. [35] and in the present work. The discussion of the effectiveness of the implemented analytical methods is beyond the scope of this work, but if the above-mentioned impurities (which are inevitably associated with the process of synthesis) are reliably detected, the need to improve implemented approaches to the synthesis of ^177^Lu-DOTA-PSMA-based radiopharmaceuticals becomes clear. As previously mentioned, the use of elevated temperatures, which provides a high radiochemical yield in a short time, is limited by the formation of thermal degradation impurities. A decrease in the synthesis temperature leads to an increase in the synthesis time required to achieve a satisfactory radiochemical yield, which in turn is limited by radiolysis. An increase in the concentration of auxiliary substances, including stabilizers-quenchers to suppress radiolysis, affects the kinetics of target complex formation and requires an increase in the synthesis temperature. The circle appears to be closed. However, one solution may be to optimize the synthesis conditions and use alternative approaches that are not yet widely used.

The most obvious parameter for optimizing the synthesis is the pH. In the synthesis of radioligands based on DOTA-conjugated molecules and lutetium-177 (as well as yttrium-90), pH of the reaction mixture of 4.0–4.5 is recommended and generally accepted [42,43]. At first glance, this fact seems counterintuitive because, with increasing pH, the degree of deprotonation of the chelating agent (DOTA) and the percentage of the deprotonated form increase, thereby increasing the kinetics of complex formation [44,45,46]. The experimental data obtained for dilute solutions with low ^177^Lu activity concentrations are consistent with this and show the possibility of achieving a high radiochemical yield with increasing pH already at ambient temperature (Figure 5). This is in good agreement, for example, with the results of the successful synthesis of ^177^Lu-labeled monoclonal antibodies, where it is necessary to maintain the reaction mixture parameters close to neutral pH, and the use of elevated temperatures is unacceptable [47,48,49]. Earlier, Kukis et al. showed, using yttrium-90 labeling of a DOTA-conjugated antibody, that increasing the pH of the reaction mixture leads to a significant increase in the reaction kinetics and high radiochemical yields in the absence of intense heating (room temperature and 37 °C, the half-times for uptake of yttrium-90 by DOTA-MAb were 46–410 min and 4.2–37 min for pH 4.5 and 6.5, respectively) [50]. Later, similar effects of pH influence were noted by Liu et al. in their works on ^90^Y and ^177^Lu labeling of a DOTA-conjugated vitronectin receptor antagonist [51,52]. However, despite the attractive kinetics of the complex formation of ^177^Lu and DOTA at elevated pH values, this process is strongly limited by the parallel hydrolysis of the radionuclide and permissible activity concentrations. The formation of the insoluble hydroxide Lu(OH)_3_ begins at pH ≥ 6.5 at C_Lu_ ≥ 10^−5^ M [53,54,55]. At activity concentrations of 1–1000 MBq/mL, the concentration of ^177^Lu (in the absence of a carrier) is 1.36–1360 nmol/L. Considering the data of López-González et al. [56] on the pM value (the limit of Lu^3+^ concentration in solution), insoluble hydroxide in the absence of a chelator at pH 7.5–8.5 will be formed at a concentration above 959 nmol/L, which corresponds to 705 MBq/mL. It is worth keeping in mind that hydrolysis processes intensify with increasing temperature: extrapolating data on the temperature dependences of the solubility products of lanthanide hydroxides [55]; it can be assumed that with an increase in temperature from 25 to 70 °C, the solubility product (*K_sp_*) will decrease by almost four orders of magnitude (Δlg*K_sp_* ≈ 3.7). This reduces the threshold pH value by approximately one unit, and when heated to 95 °C, by approximately 2.2 units. Thus, under conditions of multidose synthesis with high activity concentrations, the use of more alkaline media is fraught with the formation of radiocolloids, and the use of alternative approaches for reproducibly high radiochemical yields is clearly necessary.

The compelling advantages of microwave heating in the radiochemical synthesis of labeled compounds and, in particular, radiopharmaceuticals, have been repeatedly demonstrated in the scientific literature and thoroughly reviewed [57,58,59,60,61,62]. During the synthesis of organic molecules labeled ^3^H, ^11^C, ^18^F, and ^123^I, it has been shown that microwave heating significantly increases the yield of the final product by shortening the reaction time, without causing sample decomposition, significant degradation of the target molecule, or formation of undesirable side products, as well as improving the reproducibility of the synthesis and chemical flexibility owing to the ability to accelerate the normally sluggish reactions of weakly reactive substrates [58,59,60,61,63,64,65,66]. In particular, microwave-supported conditions have been applied to the synthesis of the most widely used PET tracer, 2-[^18^F]fluoro-2-deoxyglucose ([^18^F]FDG) [67].

Microwave-supported syntheses have been successfully used for metal-based radiopharmaceuticals as well. For obvious reasons, most of the work in this area is devoted to the synthesis of ^99m^Tc-based radiopharmaceuticals [68,69,70,71,72,73,74,75,76,77,78,79,80]. Simultaneously, the number of studies that have applied this approach to other radiometals is increasing. Microwave heating allowed to significantly reduce the reaction time, increase the yield and specific activity, and improve the reproducibility of syntheses in the case of gallium-68 and its complexes with DOTA- and NOTA-conjugated peptides (DOTA-TOC [81,82], DOTA-RDG and NODAGA-TATE [81]), oligonucleotides [81], tetrapyrrole derivatives [83,84] and bis(thiosemicarbazone) complexes [85]. The application of this approach has shown impressive results in the synthesis of various preparations with ^44^Sc ([^44^Sc]Sc-DOTA-TOC [86] and [^44^Sc]Sc-PSMA-617 [87]), ^52^Mn ([^52^Mn]Mn-porphyrins [88]), ^90^Y and ^177^Lu (the minigastrin analog CP04 [89]), and even for the synthesis of alpha-therapeutic radiopharmaceuticals with ^225^Ac [90,91,92]. Ucar implemented microwave heating in the synthesis of ^nat^La complexes with DOTA-conjugated molecules (DOTATAE and PSMA-617) to simulate ^225^Ac labeling [93].

As mentioned above, the vast majority of studies have focused on reducing the incubation time required for the efficient incorporation of radionuclides into the vector molecule and increasing the specific activity with a typical composition of excipients in the reaction mixture. Previously, on the example of [^44^Sc]Sc-PSMA-617 synthesis and the influence of oxalic acid residual content [87], we have shown that microwave heating allows leveling of the influence of interfering organic components in the reaction mixture (forming intermediate complexes with the radionuclide) and achieving the required levels of radiochemical conversion. The results obtained in this study on the synthesis of [^177^Lu]Lu-PSMA-617 with the addition of 2,3-dihydroxybenzoic acid (2,3-DHBA) confirmed this effect. 2,3-DHBA has been shown to have greater antioxidant activity than gentisic acid (2,5-DHBA, commonly used in the synthesis of radiopharmaceuticals) and other dihydroxybenzoic acids [94]. This would make it a very attractive substance for further study of the inhibition of radiolysis processes in ^177^Lu-based radiopharmaceuticals [34]. However, experimental data have shown that the direct use of 2,3-DHBA in the synthesis of ^177^Lu-preparations dramatically reduces the radiochemical yield, which is probably due to the formation of a fairly stable complex with lutetium-177 owing to the catechol group. Notably, microwave heating allows high levels of radiochemical conversion to be achieved, even under conditions where the target reaction is hampered by interfering substances. Various substances capable of interfering with the target reaction of radiopharmaceutical synthesis may be present in the reaction medium for several reasons. First, they may be introduced through the radionuclide solution owing to various features of the chromatographic or extraction isolation of this radionuclide. Second, due to the features of the reaction itself, when it is necessary to suppress the hydrolysis of the radionuclide or suppress the degradation of the vector molecule (for example, caused by radiolysis or acidity of the medium). Hence, in radiopharmaceutical technology, microwave-supported conditions make it possible to significantly expand the permissible options for synthesis in the presence of auxiliary substances of various natures and concentrations.

In the context of this study, the principal advantage of microwave heating is the significant intensification of [^177^Lu]Lu-PSMA-617 synthesis efficiency without a concomitant increase in the content of radiochemical impurities, owing to the thermocyclization and thermodegradation of the vector molecule. As can be seen from the experimental data (Figure 10), carrying out the reaction under microwave heating conditions for only one minute provides almost quantitative ^177^Lu binding (≥99%), with a radiochemical purity of ≥97%. Essentially, ~2.0% of the thermocyclization impurities determined in the sample were not formed during synthesis, but were initially contained in the precursor solution (as can be seen from the subsequently obtained results of synthesis at room temperature—Figure 13).

Finally, a significant reduction in the necessary and sufficient synthesis time under conditions of high radiation load caused by the decay of lutetium-177 (especially during multidose synthesis) provides the possibility of optimizing the composition of the reaction mixture by the required quencher content and by the maximum one-time activity of ^177^Lu used for synthesis. The analysis of the sample obtained using near-clinical lutetium-177 activity after one minute of microwave synthesis without the addition of any quenchers showed no detectable signs of radiolytic degradation of [^177^Lu]Lu-PSMA-617. In accordance with the previously established patterns of dose accumulation and the resulting drop (%) in radiochemical purity [34,95], the absorbed doses for the 4800, 7400, and 37,000 MBq of ^177^Lu would be 6, 10, and 48 Gy in the 1 min interval, respectively. The expected drop in radiochemical purity of [^177^Lu]Lu-PSMA-617 (in the absence of any quenchers) would be just ~0.7%, ~1.1%, and ~5,5%, respectively. In comparison, the dose accumulated during convection heating for the 15–30 min typical synthesis interval (with an expected drop (%) in radiochemical purity caused by radiolysis) would be 94–188 Gy (10–20%), 145–289 Gy (15–29%), and 723–1446 Gy (56–80%), respectively, for the given activity values.

The combination of the listed effects determines the possibility of synthesizing ^177^Lu-DOTA-PSMA-ligands and related compounds in the shortest possible time, not only with high radiochemical conversion (labeling yield), but also with high radiochemical purity, considering both the impurity of the unbound radionuclide and the impurities of thermal and radiolytic degradation of the vector molecule. Despite the active development of microwave heating methods in radiopharmaceutical technology, this approach is not yet generally accepted or easily accessible. Additional difficulties may arise when implementing this method in the existing synthesis schemes that use automated modules. In such cases, an alternative approach is possible.

Mixed media with the addition of an organic solvent to the reaction mixture are also actively used in radiopharmaceutical technology. Eppard et al. showed that adding ethanol to the reaction mixture significantly improved DOTATOC labeling with ^68^Ga and ^44^Sc in terms of the temperature, time, and concentration of the precursor [96,97].

Kersemans et al. showed that the use of ethanol improves the labeling yield of Al[^18^F]PSMA-11 from ~60% to ~85%, with a corresponding increase in the ethanol fraction in the reaction mixture from 0 to 50–60 vol.% [98]. Imura et al. showed that the use of DMSO (in comparison with methanol, ethanol, and dimethylformamide) allows to improve the radiochemical yield of the [^89^Zr]Zr-PSMA-617 synthesis (50 vol.% of organic solvent, 30 min at 90 °C) [99].

Pérez-Malo et al. carried out the study to assess the effect of adding an organic solvent (ethanol, isopropanol, and acetonitrile) on the efficiency of complex formation between DOTA and ^44^Sc, ^68^Ga, and ^177^Lu radionuclides [100]. In the case of lutetium-177 the study was carried out in 0.1 M sodium acetate buffer (pH 8), [Lu]:[DOTA] ratio of 1:10, 70 °C and 0−30 vol.% of EtOH. After 30 min of incubation, the radiochemical yield of [^177^Lu]Lu-DOTA was approximately 70 ± 8% with the addition of 10–30 vol.% ethanol (according to the presented dependence graph, with no significant difference for alcohol fractions) and around 35 ± 7% without ethanol. The authors claimed that hydration/solvation of the radionuclide (III) ion does not play an important role in the formation rate of the final [M(DOTA)]^−^ complexes, and the rate-controlling step is the H_2_O- and OH^−^-assisted deprotonation and rearrangement of the monoprotonated [M(HDOTA)] intermediates formed in fast equilibrium with the diprotonated [M(H_2_DOTA)]^+^ intermediates. Thus, ethanol indirectly accelerates the formation of the final [M(DOTA)]^−^ complex by decreasing the protonation constant (K_M(HL)_^H^) of the monoprotonated [M(HDOTA)] intermediates. However, we believe that considering the effects described in the literature as well as in the present work, it is necessary to additionally consider the hydrolysis/solvation of the radionuclide cation in a mixed medium (taking into account that [100] also presents data from NMR studies indicating a significant change in the solvation shell of cations). It has been shown [101] that when the chemical form of the cation changes from a hydrate ([M(H_2_O)_n_]^3+^) to a chelate ([ML(H_2_O)_m_]^3+^) containing water in the inner sphere, the rate constant of the exchange of solvent molecules between the inner and outer coordination spheres drops significantly (up to two orders of magnitude). This, in turn, can significantly affect both the protonation/deprotonation of the resulting complex and the rate of equilibrium between all the forms. The introduction of an organic solvent into a solution reduces the water content, water activity, and dielectric permittivity of the solution in general. For example, the effect of a decrease in the dielectric permittivity of mixtures of water with methanol and ethanol on the increase in the complexation constants was noted in [102,103,104,105]. Aqua complexes of ions are solvated in solution owing to electrostatic hydration and van der Waals interactions between the water (or other molecules) of the solution and the water of the first coordination sphere of the cation. This led to the existence of contact and solvate-separated ion pairs. The gradual replacement of water molecules with less polar solvent molecules, both in the outer solvation shell and in the inner coordination sphere, leads to a weaker screening of the cation charge, in general, and to the loss of symmetry of the solvation complex, in particular [106,107,108,109]. This, in turn, can lead to an increase in the reactivity of the cation. Thus, it is possible to explain, for example, the change in the sorption behavior of gallium-68 in water-ethanol and water-acetone media [110,111]. Another important consequence of introducing an organic solvent into an aqueous solution is a change in the autoprotolysis constant of the solvent [112]. However, measuring the potential of hydrogen in mixed media is a non-trivial task without a generally accepted solution [113,114]. When measuring the pH of ethanol-containing mixtures (80%) using a combined electrode calibrated using standard buffer solutions (in an aqueous medium), a shift in the value by approximately two units may be observed relative to an ethanol-free solution. This shift cannot be attributed to a real change in the acidity level. Moreover, a number of studies have shown that, in reality, the difference between the “reduced” pH of the mixture and the pH of the aqueous solution is no more than 1 unit [115,116,117,118]. A detailed consideration of this issue is beyond the scope of this study; however, we believe that the effect of changing the pH when moving to a mixed mixture in the case of binding trivalent metals with a chelator, such as DOTA, is not as significant as the change in solvation shells. In turn, the refinement and improvement of the thermodynamic model of the influence of an organic solvent on the kinetics of formation of the final radionuclide-ligand complex, taking into account both the parameters of deprotonation of intermediate forms and the processes of solvation and hydrolysis of the cation under conditions of changing autoprotolysis of the solvent, is a matter for further research.

In general, our results are consistent with previously published data on the use of ethanol additives during radiochemical synthesis and its positive influence. In the context of the main topic of this work, the addition of ethanol significantly reduced the temperature and time of synthesis, achieving satisfactory radiochemical yields while minimizing the content of impurities caused by thermal degradation of the PSMA-ligands pharmacophore. It is noteworthy that, in the work of Pérez-Malo et al. [100], the yield of the complex formation of [^177^Lu]Lu-DOTA reaches the same level for the same incubation time regardless of the alcohol fraction studied (10–30 vol.%). This effect echoes the data of [96], where the authors stated that the positive effects of ethanol reach saturation at a content of 40%. Interestingly, however, the results described do not correlate with our data, even though the authors [100] performed the synthesis under suboptimal conditions. Experimental data showed a clear positive dependence of the kinetics of the [^177^Lu]Lu-PSMA-617 synthesis reaction on each ethanol fraction. Depending on other parameters of the reaction mixture (volume, buffer concentration, amount of precursor (metal-to-ligand), and presence of additional auxiliary substances (e.g., gentisic acid)), the optimum ethanol concentration for obtaining a satisfactory yield in the shortest time will vary. Therefore, it is fair to speak only about a conditional optimum and not about any saturation of the positive effect in general. Thus, if it is possible to realize the synthesis using a dilute buffer (about 0.03 M sodium acetate, if the characteristics of the initial radionuclide solution allow), the almost quantitative yield can be obtained in 10–15 min already at room temperature with the addition of 40 vol.% ethanol.

Currently, the allowed dose of ethanol for parenteral preparations is 0.5 vol.%, if considered as residual solvent. However, its content may be significantly higher if ethanol is used as the excipient in the finished dosage form. Several injectable antineoplastic drugs have been formulated using ethanol [119]. According to the European Medicines Agency, the threshold for ethanol content is set at 3 g per administration [120]. However, some cytotoxic drugs in their commercial formulations may contain even more than 3 g of ethanol as an excipient [119]. In the case of radiopharmaceuticals, ethanol is commonly used as an additive in different formulations, with a typical and generally accepted concentration of ≤10 vol.% [121]. According to the *Guide for the Elaboration of Monographs on Radiopharmaceutical Preparations*, ethanol content should be a maximum of 10 vol.% and a maximum of 2.5 g per administration, taking the density to be 0.790 g/mL [122]. For the approaches considered, assuming a single-dose synthesis in a 5 mL reaction mixture containing 40 vol.% ethanol, the mass of ethanol would be 1.58 g (within the recommended limits). Dilution by a factor of 4 will result in 20 mL of the final formulation, with an allowable concentration of 10 vol.%. If the amount of lutetium-177 activity in such a reaction mixture corresponds to two or more clinical doses, subsequent dilution of the preparation for individual patient doses will result in even lower ethanol content. Thus, the proposed synthesis approach is well within the established ethanol content limits and will not cause any difficulties or limitations for clinical application.

Additionally, performing test syntheses with the analytical activities of lutetium-177 under the conditions described at room temperature can be a convenient and effective test of precursor incoming control for the initial content of thermocyclization impurities (see Supplementary Materials Figure S14). Changing the buffer concentration and the other parameters of the reaction mixture requires changing the ethanol fraction to achieve comparable results. Hence, the best approach does not seem to be a strict recommendation for any specific synthesis conditions, but optimization of already available techniques by selecting the necessary and sufficient alcohol fraction to achieve satisfactory reaction yields and high radiochemical purity at reduced temperatures (see Supplementary Materials Figures S15 and S16).

The kinetics of ^177^Lu incorporation into the structure of DOTA-conjugated molecules are mostly determined by the properties of the chelating agent (DOTA) rather than the rest of the conjugate (at least in the case of low molecular weight compounds with sufficient water solubility). Therefore, the proposed conditions are very likely to be applicable to other DOTA-conjugated PSMA-ligands. In confirmation of this fact, the proposed approaches have shown efficiency in the case of new molecules (kindly synthesized and provided by Dr. Aleksei Machulkin [123]—Figure S17). During the synthesis of radioconjugates of these molecules with lutetium-177, both the characteristic formation of thermocyclization impurities and the possibility of achieving a high radiochemical yield and radiochemical purity at room temperature by adding ethanol (up to 40%) to the reaction mixture were observed (see Supplementary Materials Figures S18 and S19).

## 5. Conclusions

The improvement in synthesis methods that minimize the formation of possible radiochemical impurities (in the context of recent studies) while maintaining satisfactory radiochemical yields is an integral part of the implementation of high-tech nuclear medicine procedures at the modern level. The results of this study, in conjunction with previously published data, strongly suggest that microwave heating and ethanol addition ensure high radiochemical yield and radiochemical purity in the shortest possible time and at the minimum necessary and sufficient synthesis temperature. Both approaches are viable for further implementation in the clinical practice of ^177^Lu-PSMA radiopharmaceutical manufacturing. It is important to note that in the synthesis of PSMA-617 radioconjugates (and similar molecules) with other medical radionuclides, synthesis protocols that involve the use of high temperatures and/or long incubation times have been described, often due to the peculiarities of complex formation between the chelating agent and the radionuclide. For example, [^18^F]F-PSMA-617-NODA—15 min at 110 °C [38], [^89^Zr]Zr-PSMA-617 and [^89^Zr]Zr-PSMA^I&T^—30 min at 95 °C [124] and 90 min at 95 °C [125], [^225^Ac]Ac-PSMA-617—40–50 min at 120 °C [126], etc. Obviously, the drop in real radiochemical purity owing to the formation of thermal degradation impurities should not be neglected in favor of only a high radiochemical yield of synthesis. From that point of view, it is reasonable to assume that the approaches described may be applied to the synthesis of various PSMA-ligands and related compounds (especially those that are sensitive to heat) with lutetium-177 and other medical radionuclides as well. However, this conclusion should be validated in future studies.

## Figures and Tables

**Table 1:** Radio-TLC methods used to analyze the radiochemical conversion of [^177^Lu]Lu-PSMA-617 preparations.

#	Stationary Phase	Solvent	*R_f_* of ^177^Lu Species ^1^	
			Unbound [^177^Lu]Lu^3+^	[^177^Lu]Lu-PSMA-617
1	TLC Silica gel 60 sheets with aluminum support (5553, Merck, Darmstadt, Germany)	MeCN–H_2_O (1:1)	0–0.05	0.85 ± 0.05
2	iTLC-SG glass microfiber chromatography paper impregnated with a silica gel (Agilent, Santa Clara, CA, USA)	0.05 M H_3_Citr_aq_	0.95 ± 0.05	0.15 ± 0.05
3		NH_3_–Ethanol–H_2_O (1:5:10)	0–0.05	0.95 ± 0.05
4		1 M CH_3_COONH_4aq_–CH_3_OH (1:1)	0.95 ± 0.05 ^2^	0.95 ± 0.05

^1^ Naturally, lutetium-177 radiocolloids, if present, have a retardation factor of 0 in all TLC systems. ^2^ Tailing peak along the entire length of the strip.

**Table 2:** Radio-HPLC methods used to analyze the radiochemical purity of [^177^Lu]Lu-PSMA-617 preparations.

#	HPLC Column	Gradient Profile, Flow Rate	*R_t_* of ^177^Lu Species, min			Based on Method from
			Unbound [^177^Lu]Lu^3+^	[^177^Lu]Lu-PSMA-617	[^177^Lu]Lu-PSMA-617 Thermodegradation Products	
1	Phenomenex Luna^®^ 250 × ⌀4 mm, 5 μm, 100 Å	0-1-25-27-29-32 min = 5-5-95-95-5-5% B, 1 mL/min	2.45 ± 0.05	10.85 ± 0.05	11.0–11.5	[35]
2	Phenomenex Luna^®^ 150 × ⌀4 mm, 5 μm, 100 Å	0-5-15-20 min = 17-25-25-17% B, 0.75 mL/min	1.6 ± 0.1	8.41 ± 0.02	7.0 ± 0.1 9.05 ± 0.05 9.47 ± 0.03 10.13 ± 0.6	[34,39]

## Data Availability

The data presented in this study are available upon request from the corresponding author.

## Supplementary Materials

The following supporting information can be downloaded at https://www.mdpi.com/article/10.3390/pharmaceutics16121535/s1, Figure S1: Radio-HPLC chromatograms of the model sample [^177^Lu]Lu-PSMA-617 obtained with methods 1 and 2; Figure S2: Result of UPLC-MS analysis (MS ESI(+) TIC, *m*/*z* 50-2000) of [^177^Lu]Lu-PSMA-617 sample obtained by prolonged intensive heating for 2 h at 120 °C (Figure 3); Figure S3: Result of UPLC-UV analysis (MS conditions) of [^177^Lu]Lu-PSMA-617 sample obtained by prolonged intensive heating for 2 h at 120 °C (Figure 3); Figure S4: Fragmentation scheme of PSMA-617; Figure S5: MS^E^ spectra of cleavage product of the Glu-CO fragment (*R_t_* 9.81 min Figure S2); Figure S6: MS^E^ spectra of PSMA-617 (*R_t_* 11.25 min Figure S2); Figure S7: MS^E^ spectra of dehydrated (hydantoin-containing) PSMA-617 (*R_t_* 11.62 min Figure S2); Figure S8: MS^E^ spectra of dehydrated (hydantoin-containing) PSMA-617 (*R_t_* 11.75 min Figure S2); Figure S9: MS^E^ spectra of dehydrated (pyroglutamate-containing) PSMA-617 (*R_t_* 11.98 min Figure S2); Figure S10: Dependence of the radiochemical yield of [^177^Lu]Lu-PSMA-617 with addition of gentisic acid (GA) on synthesis temperature; Figure S11: Dependence of the radiochemical yield of [^177^Lu]Lu-PSMA-617 on ethanol content in the reaction mixture; Figure S12: Dependence of the ^177^Lu(III) distribution coefficients (*D_g_*) on ethanol content for Sep-Pak Accell Plus CM cation exchange sorbent (0.25 M sodium acetate, pH 4.5); Figure S13: Kinetics of [^177^Lu]Lu-PSMA-617 formation at different metal-to-ligand mole ratio; Figure S14: Radio-chromatogram (Method 2) of the [^177^Lu]Lu-PSMA-617 preparation with analytical activity of lutetium-177 (test synthesis for a precursor incoming control); Figure S15: The kinetics of the [^177^Lu]Lu-PSMA-617 synthesis with and without the addition of gentisic acid (GA, 10 mg) and ethanol (20 vol.%); Figure S16: Radio-chromatogram (Method 2) of the test [^177^Lu]Lu-PSMA-617 preparation with clinical activity; Figure S17: Chemical structures of two new PSMA ligands used as an example; Figure S18: Radio-HPLC chromatograms of the ^177^Lu-radioconjugates with new PSMA ligands synthesized at different conditions; Figure S19: Kinetics of [^177^Lu]Lu-radioconjugates formation in the presence and absence of ethanol in comparison with [^177^Lu]Lu-PSMA-617 formation kinetics.
