# Supplementary material for: Improvement of End-of-Synthesis Radiochemical Purity of 177Lu-DOTA-PSMA-Ligands with Alternative Synthesis Approaches: Conversion Upswing and Side-Products Minimization
Source: Pharmaceutics. 2024 Nov 30;16(12):1535. doi: 10.3390/pharmaceutics16121535 (PMC11677419; doi:10.3390/pharmaceutics16121535)
Supplement: Supplementary file 1 [file pharmaceutics-16-01535-s001.zip › pharmaceutics-3321897-supplementary.pdf]

pharmaceutics

Supplementary Materials to

Improvement of End-of-Synthesis Radiochemical Purity of ^{177}Lu-DOTA-PSMA-ligands with Alternative Synthesis Approaches: Conversion Upswing and Side-Products Minimization

Anton Larenkov †,*, Iurii Mitrofanov † and Marat Rakhimov

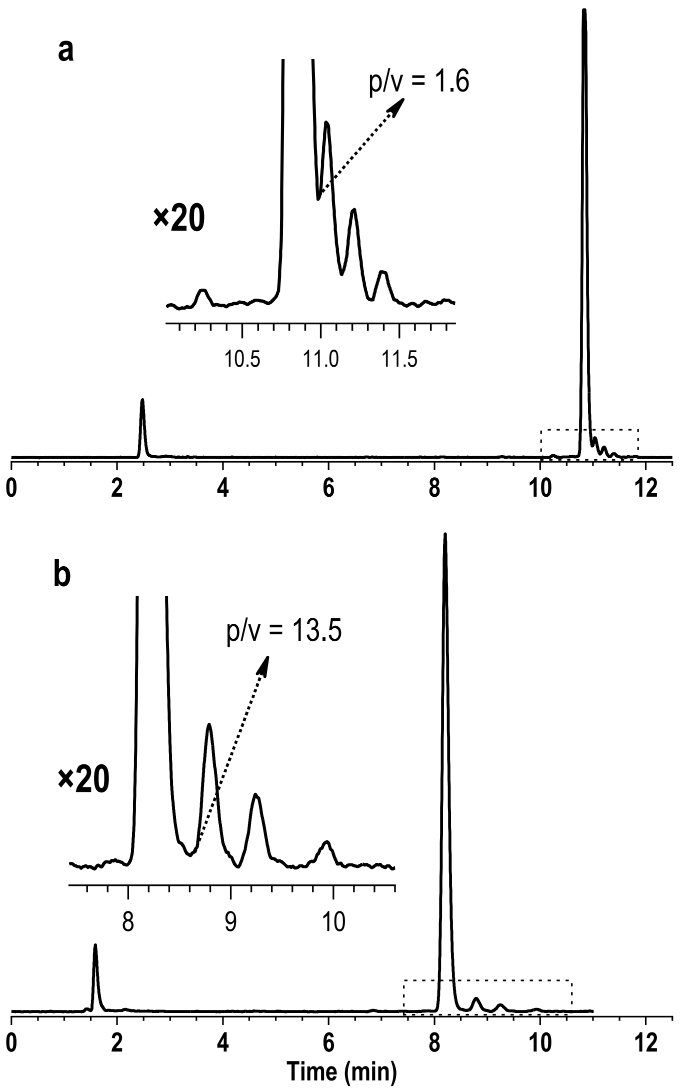

Figure S1. Radio-HPLC chromatograms of the model sample [^{177}Lu]Lu-PSMA-617 synthesized at 95 °C for 15 min (with the addition of 10% free [^{177}Lu]Lu^{III}), obtained with methods 1 and 2.

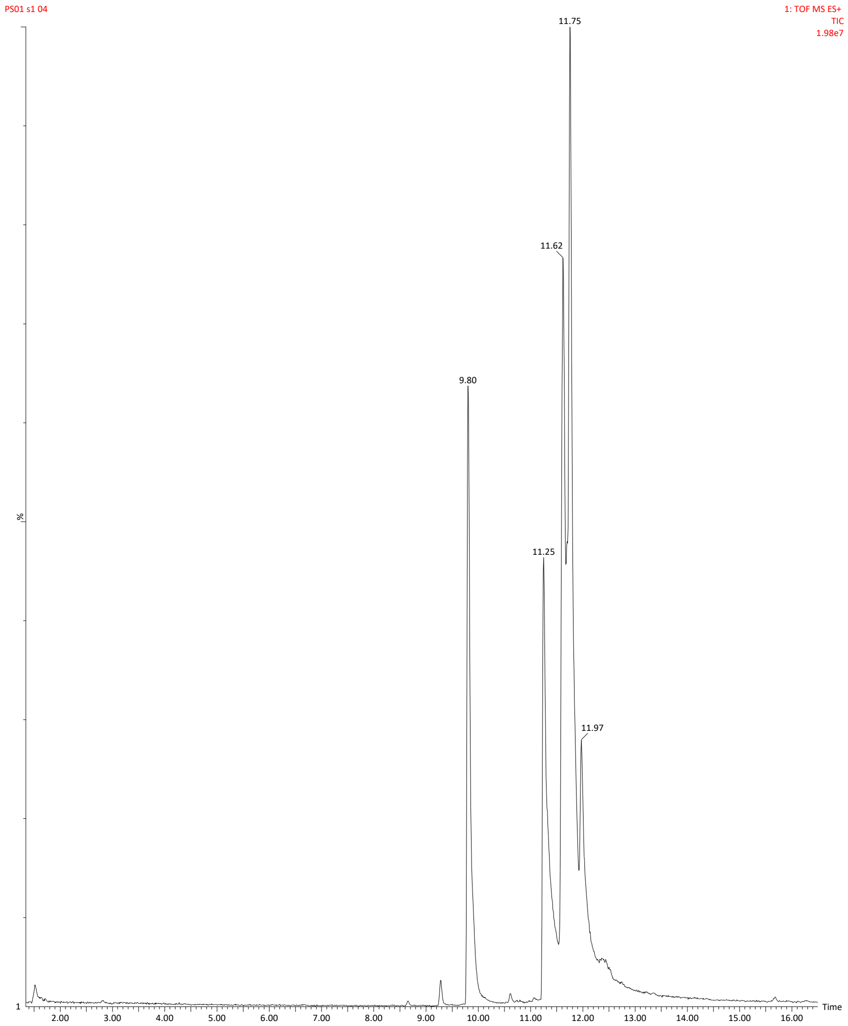

Figure S2. Result of UPLC-MS analysis (MS ESI(+) TIC, m/z 50–2000) of [^{177}Lu]Lu-PSMA-617 sample obtained by prolonged intensive heating for 2 h at 120 °C (**Figure 3**).

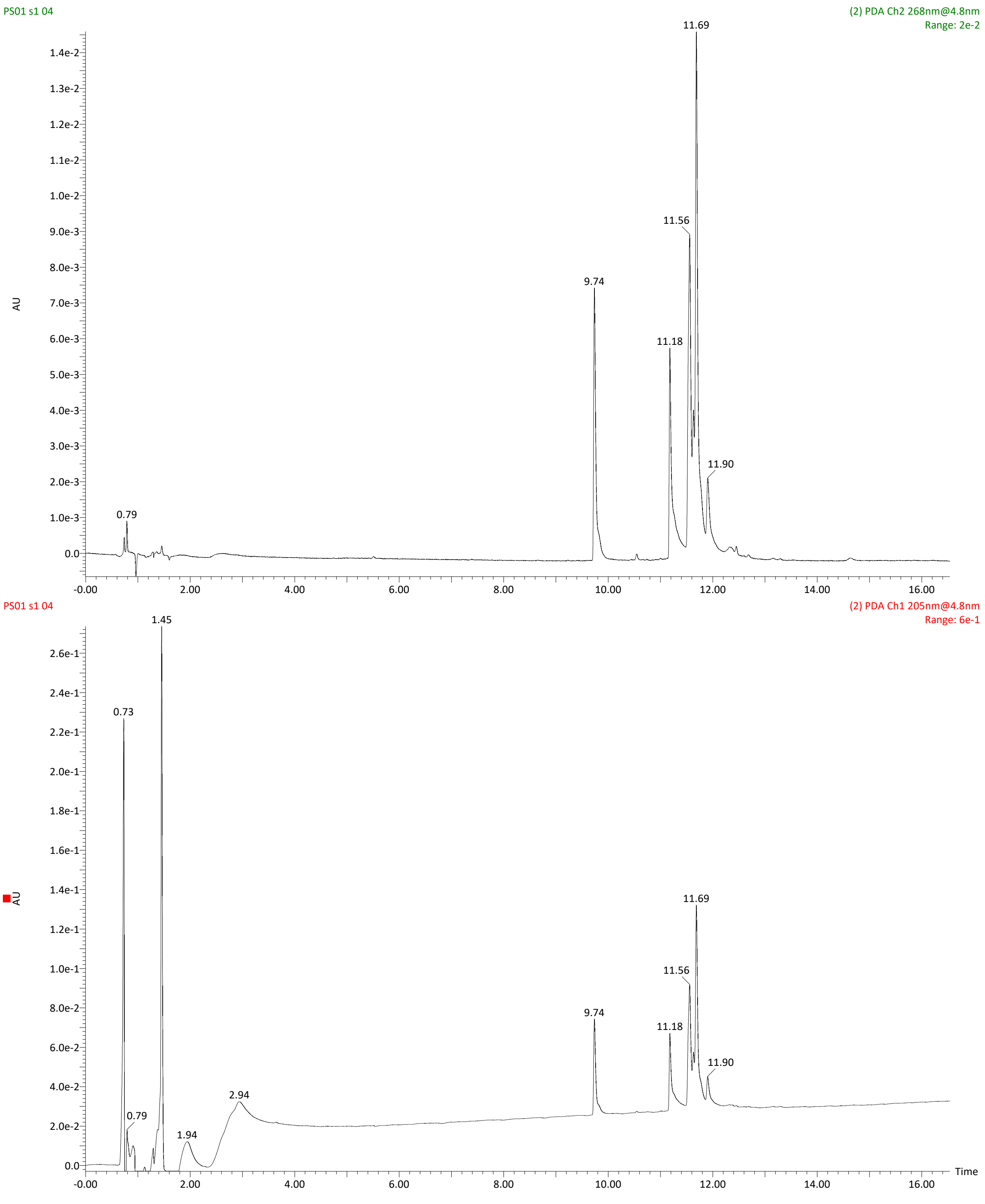

Figure S3. Result of UPLC-UV analysis (MS conditions) of [^{177}Lu]Lu-PSMA-617 sample obtained by prolonged intensive heating for 2 h at 120 °C (**Figure 3**): 268 nm (upper) and 205 nm (lower).

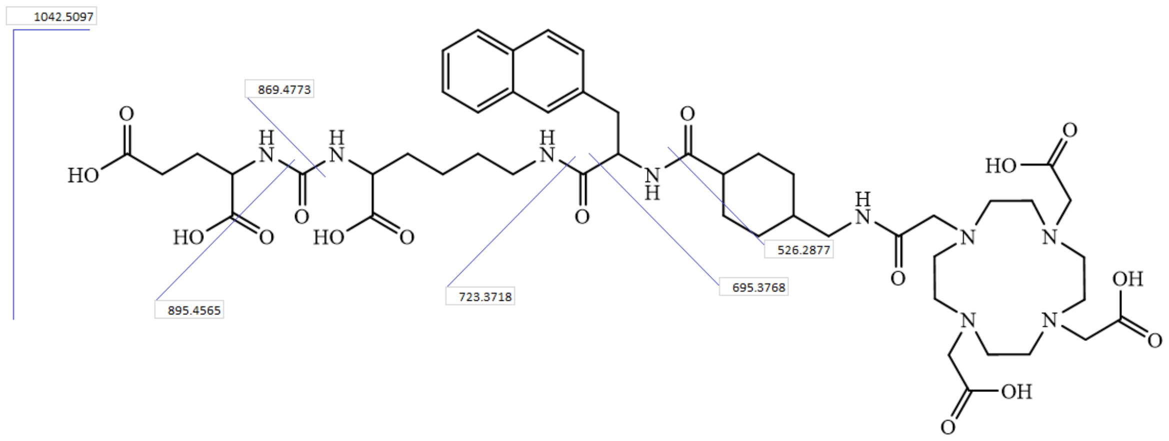

Figure S4. Fragmentation scheme of PSMA-617.

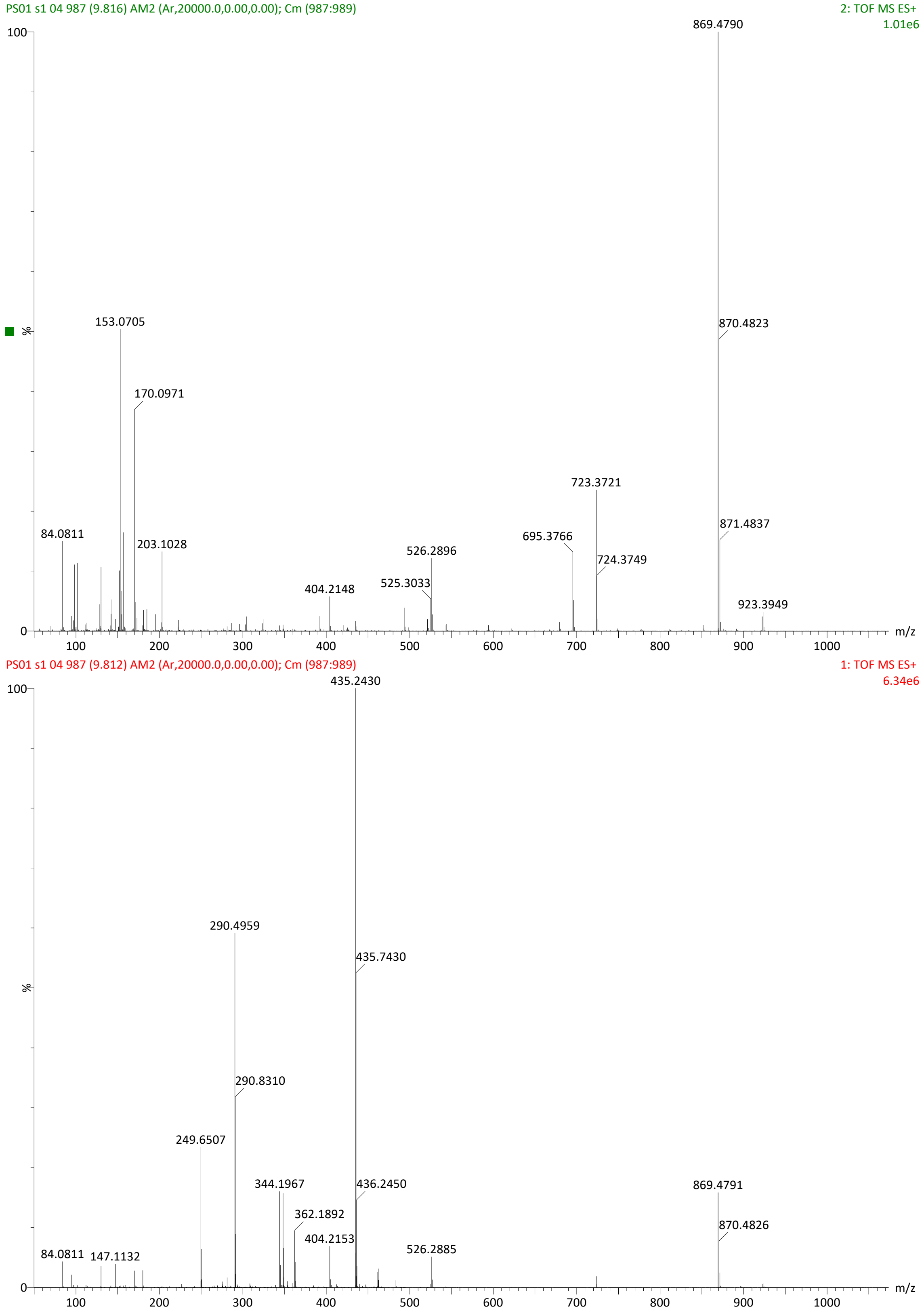

Figure S5. MS^E spectra of cleavage product of the Glu-CO fragment (*Rt* 9.81 min **Fig. S2**) at low energy regime (top) and in high energy regime (bottom).

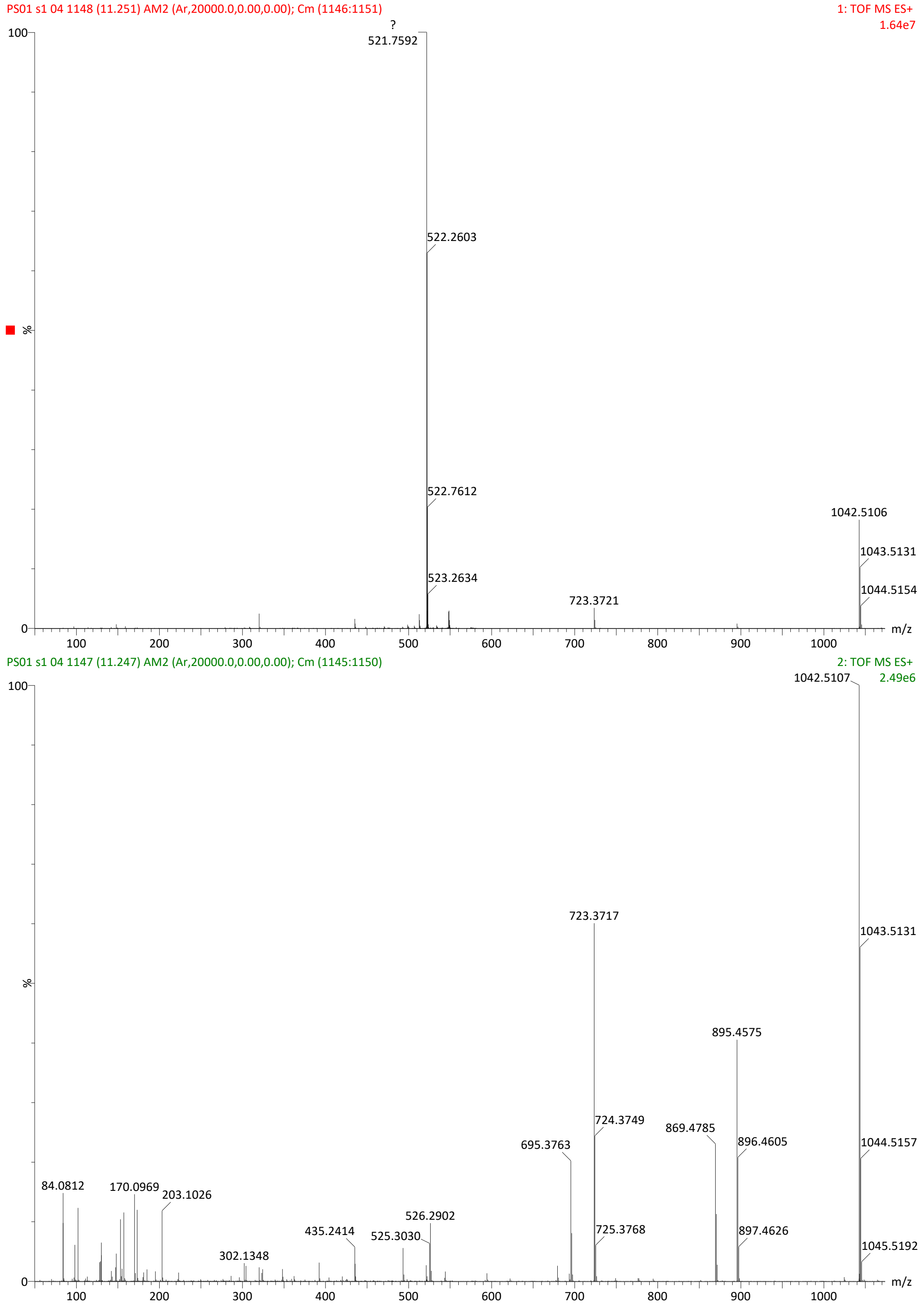

Figure S6. MS^E spectra of PSMA-617 (*Rt* 11.25 min **Fig. S2**) at low energy regime (top) and in high energy regime (bottom).

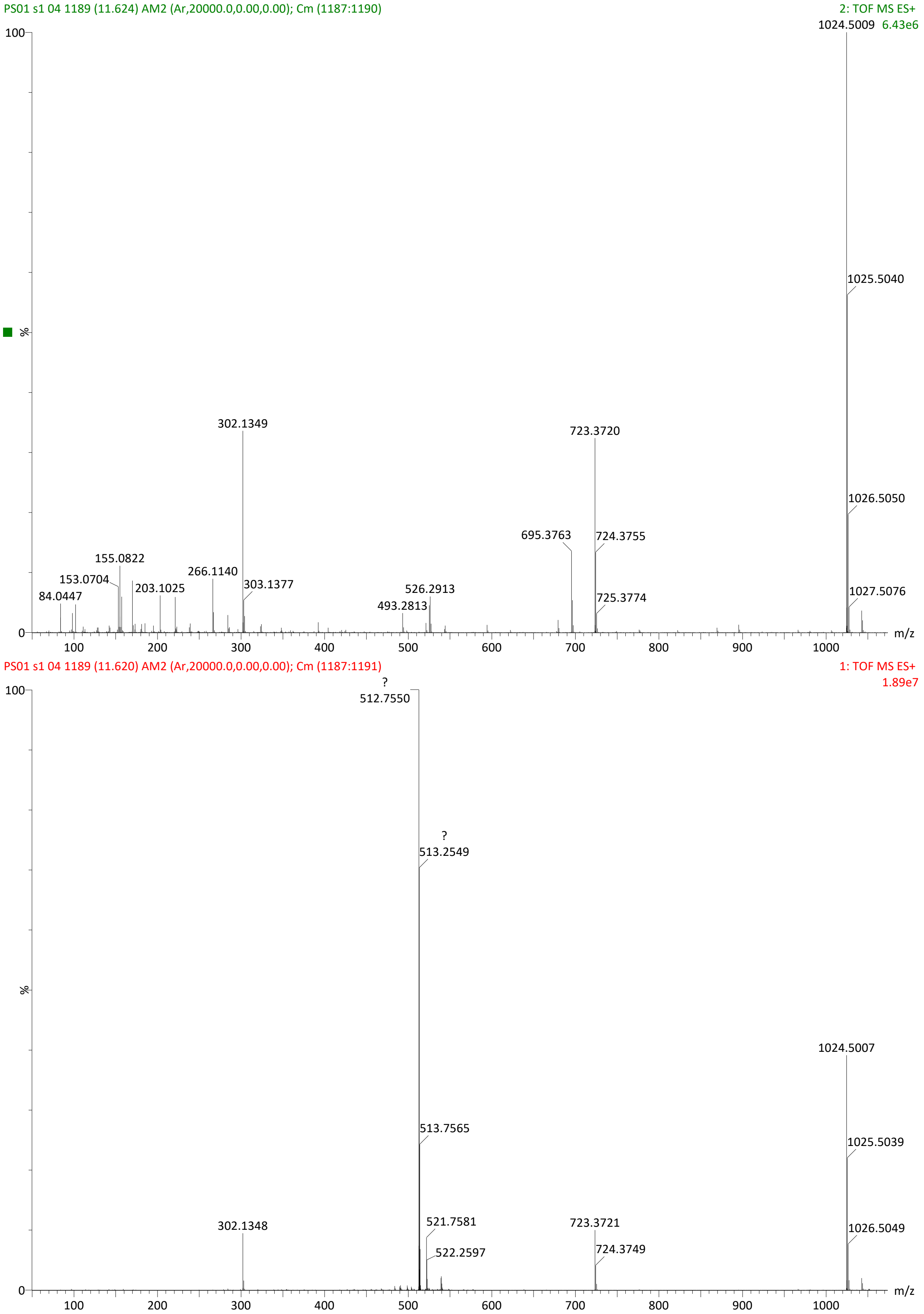

Figure S7. MS^E spectra of dehydrated (hydantoin-containing) PSMA-617 (*Rt* 11.62 min **Fig. S2**) at low energy regime (top) and in high energy regime (bottom).

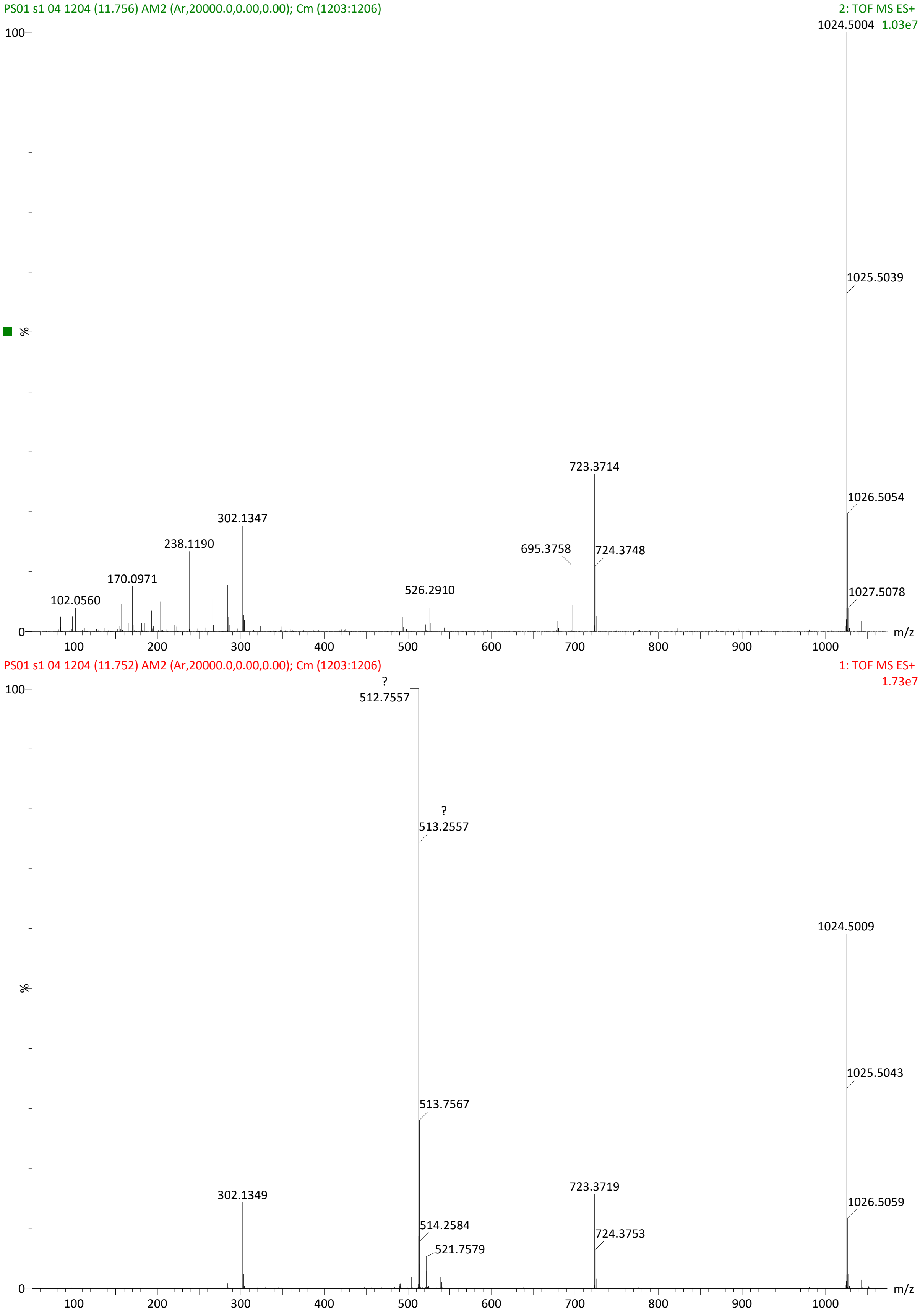

Figure S8. MS^E spectra of dehydrated (hydantoin-containing) PSMA-617 (*Rt* 11.75 min **Fig. S2**) at low energy regime (top) and in high energy regime (bottom).

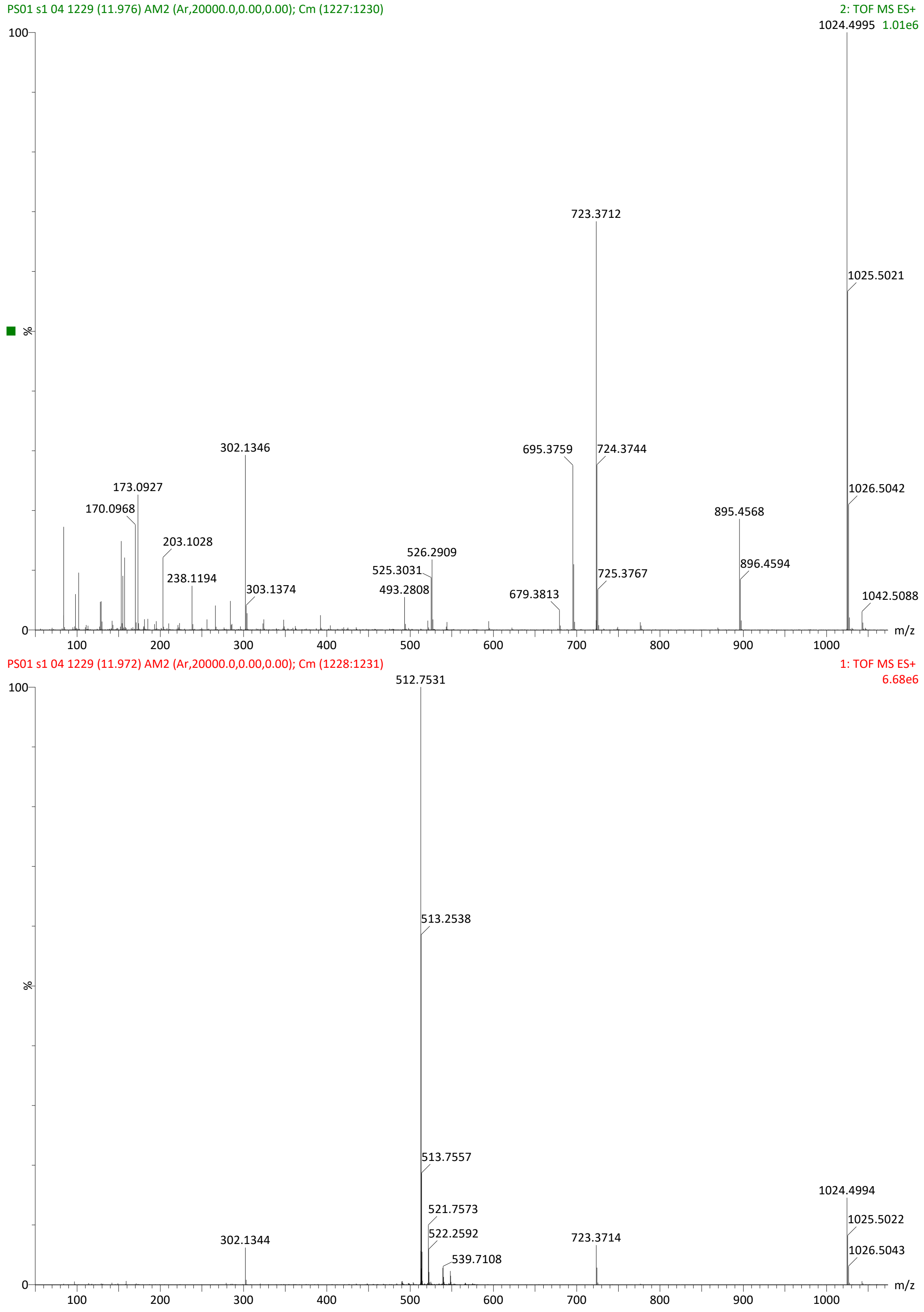

Figure S9. MS^E spectra of dehydrated (pyroglutamate-containing) PSMA-617 (*Rt* 11.98 min **Fig. S2**) at low energy regime (top) and in high energy regime (bottom).

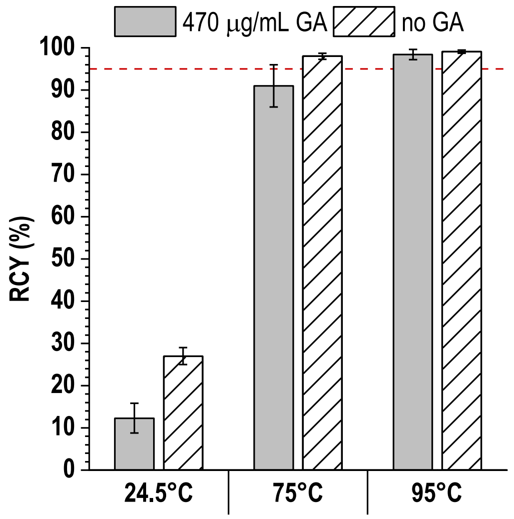

Figure S10. Dependence of the radiochemical yield of [^{177}Lu]Lu-PSMA-617 with addition of gentisic acid (GA) on synthesis temperature (incubation time — 5 min). The volume of the reaction mixture was 1 mL, the amount of PSMA-617 was 20 μg, the activity of ^{177}Lu was 250 MBq, 0.03 M sodium acetate (pH 4.5), $^{nat}LuCl_3$ added, [Lu]:[PSMA] = 1:10. RCY values are presented as mean ± SD, n = 3.

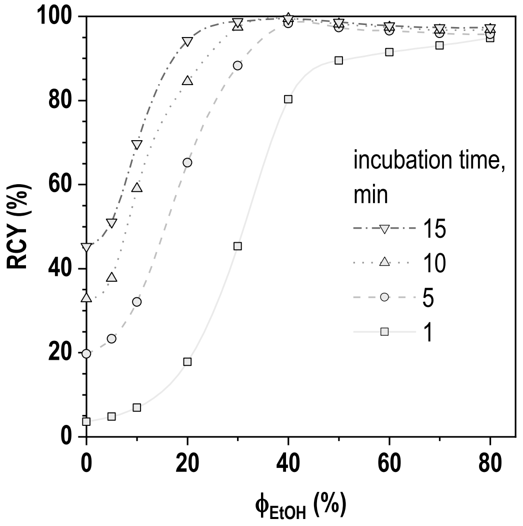

Figure S11. Dependence of the radiochemical yield of [^{177}Lu]Lu-PSMA-617 on ethanol content in the reaction mixture (for different incubation times at ambient temperature). The volume of the reaction mixture was 1 mL, the amount of PSMA-617 was 20 μg, the activity of ^{177}Lu was 250 MBq, 0.03 M sodium acetate (pH 4.5), $^{nat}LuCl_3$ added, [Lu]:[PSMA] = 1:10. The RCY values are presented as the mean for n = 3 (error bars are omitted for clarity).

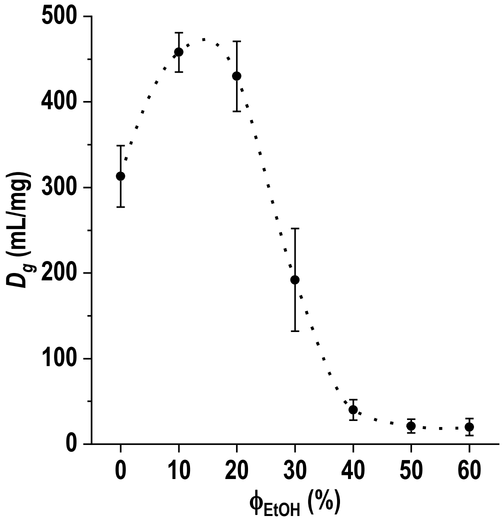

Figure S12. Dependence of the ^{177}Lu(III) distribution coefficients (D_g) on ethanol content for Sep-Pak Accell Plus CM cation exchange sorbent (0.25 M sodium acetate, pH 4.5).

The mass distribution coefficient D_g was measured by the batch static method with equilibration of a known volume (1 mL) of radionuclide spiked solution (~100 MBq of ^{177}Lu) of appropriate concentration (0.25 M sodium acetate, pH 4.5, 0–60 vol.% of ethanol) with known weight of CM sorbent (typically 15 mg). Resins were weighed and used without preconditioning. The samples were mixed in the mechanical mixer with temperature control Bioer Mixing block MB-102 (Bioer, Hangzhou, China) for 24 h at 20 °C with stirring speed 1350 rpm. After that, the samples were centrifuged (Heidolph, Schwabach, Germany) for one minute at 15,000 rpm. The tubes were removed carefully (to avoid phase mixing). Then, the aliquots (100–500 µL) of every solution were taken and their relative activities were measured. The mass distribution coefficient (mL/g) was defined according to the following equation:

$$D_g = \frac{A_0 - A}{A} \times {}^{V_{mL}}/_{m_g},$$

where A_0—count rate of the solution aliquot before the contact with the resin (decay corrected); A—count rate of the solution aliquot after the contact with the resin (decay corrected); V—solution volume, mL; m—weight of dry resin, g. Every D_g coefficient was determined at least in 5 parallel experiments.

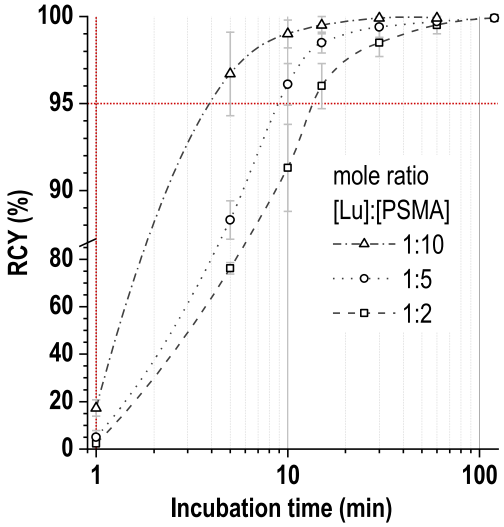

Figure S13. Kinetics of [^{177}Lu]Lu-PSMA-617 formation at different metal-to-ligand mole ratio. The volume of the reaction mixture was 1 mL, the amount of PSMA-617 was 20 μg, the activity of ^{177}Lu was 250 MBq, 0.25 M sodium acetate (pH 4.5), $^{nat}LuCl_3$ added, ethanol fraction was 20 vol.%, incubation at 60 °C. RCY values are presented as mean ± SD, n = 3

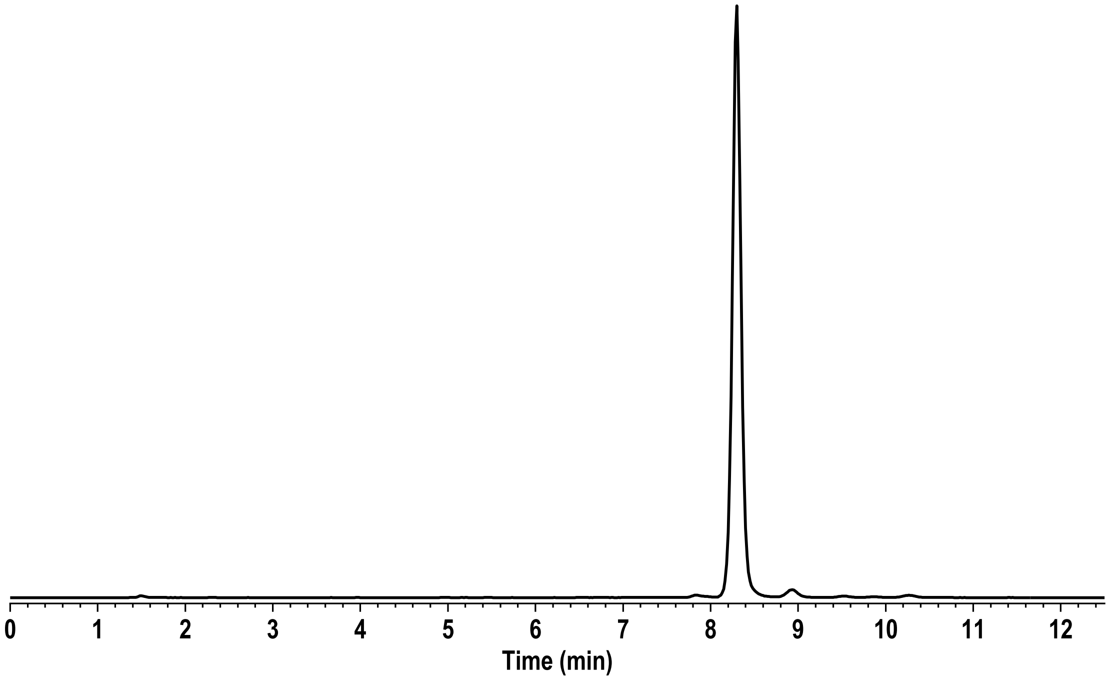

Figure S14. Radio-chromatogram (Method 2) of the [^{177}Lu]Lu-PSMA-617 preparation with analytical activity of lutetium-177 (test synthesis for a precursor incoming control). The volume of the reaction mixture was 1 mL, the amount of PSMA-617 was 20 μg, the activity of ^{177}Lu was 250 MBq, 0.03 M sodium acetate (pH 4.5), ethanol fraction was 40 vol.%, 10 min incubation at room temperature. The amount of the initial thermodegradation impurities observed in precursor was ~3.5%.

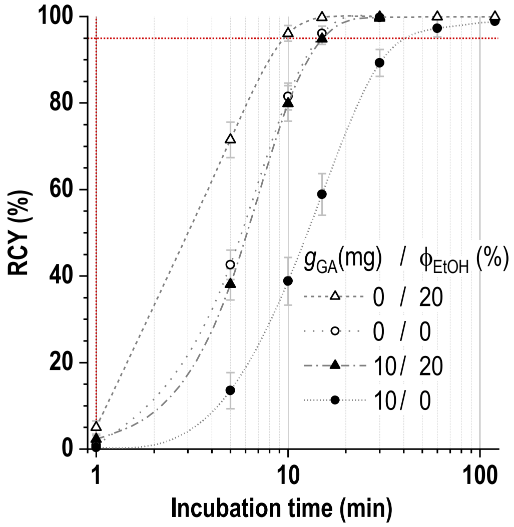

Figure S15. The kinetics of the [^{177}Lu]Lu-PSMA-617 synthesis with and without the addition of gentisic acid (GA, 10 mg) and ethanol (20 vol.%). The main synthesis parameters (reaction mixture volume, buffer concentration, metal-to-ligand ratio, and amount of gentisic acid) were adopted from the existing synthesis techniques in the clinical department. The volume of the reaction mixture was 3.56 mL, the amount of PSMA-617 was 20 μg, the activity of ^{177}Lu was 250 MBq, 0.1 M sodium ascorbate (pH 4.5), $^{nat}LuCl_3$ added, [Lu]:[PSMA] = 1:5, incubation at 60 °C. RCY values are presented as mean ± SD, n = 3.

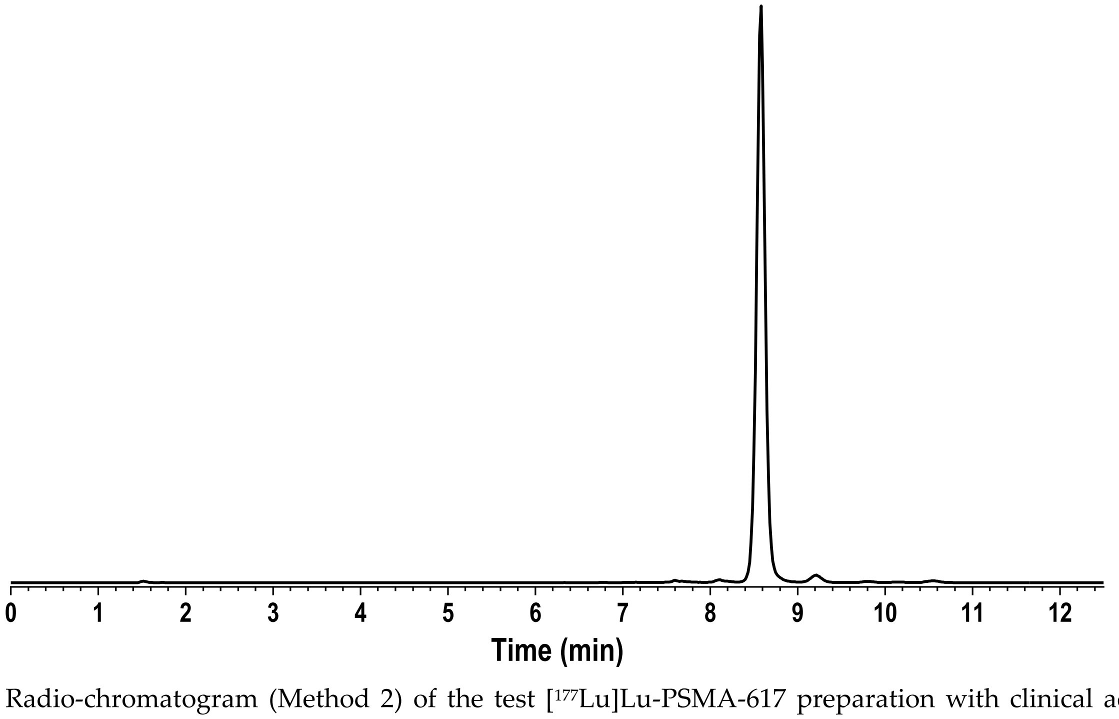

Figure S16. Radio-chromatogram (Method 2) of the test [^{177}Lu]Lu-PSMA-617 preparation with clinical activity. The volume of the reaction mixture was 3.56 mL, the amount of PSMA-617 was 390 μg, the activity of ^{177}Lu was 57 GBq, 0.1 M sodium ascorbate (pH 4.5), ethanol fraction was 20 vol.%, 10 min incubation at 60 °C. Total radiochemical yield (with CM cartridge purification) was 98.7%. The amount of thermodegradation impurities was 3.8% (initial content – 3.5%, **Figure S14**). RCP — 95.8%

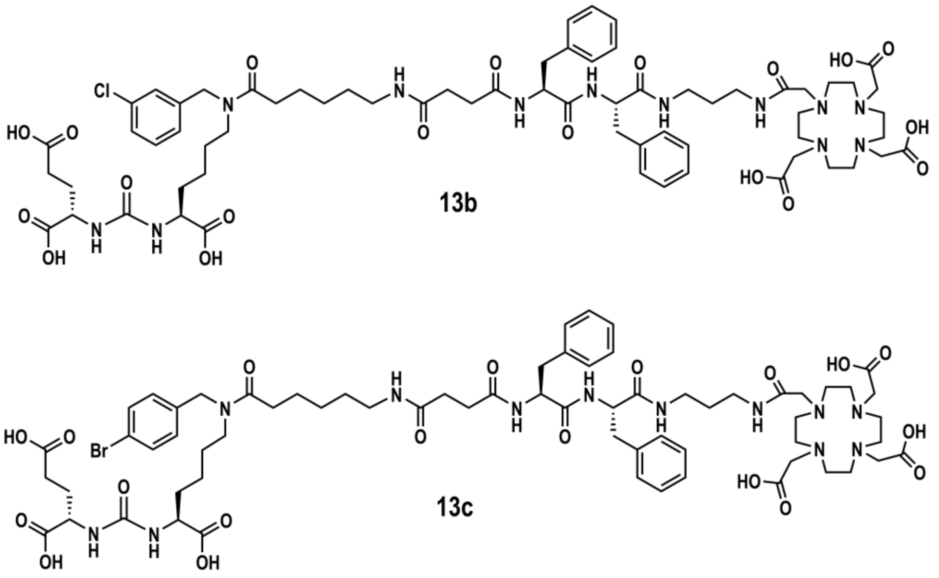

Figure S17. Chemical structures of PSMA ligands synthesized and kindly provided by Dr. Aleksey Machulkin (M.V. Lomonosov Moscow State University) [1].

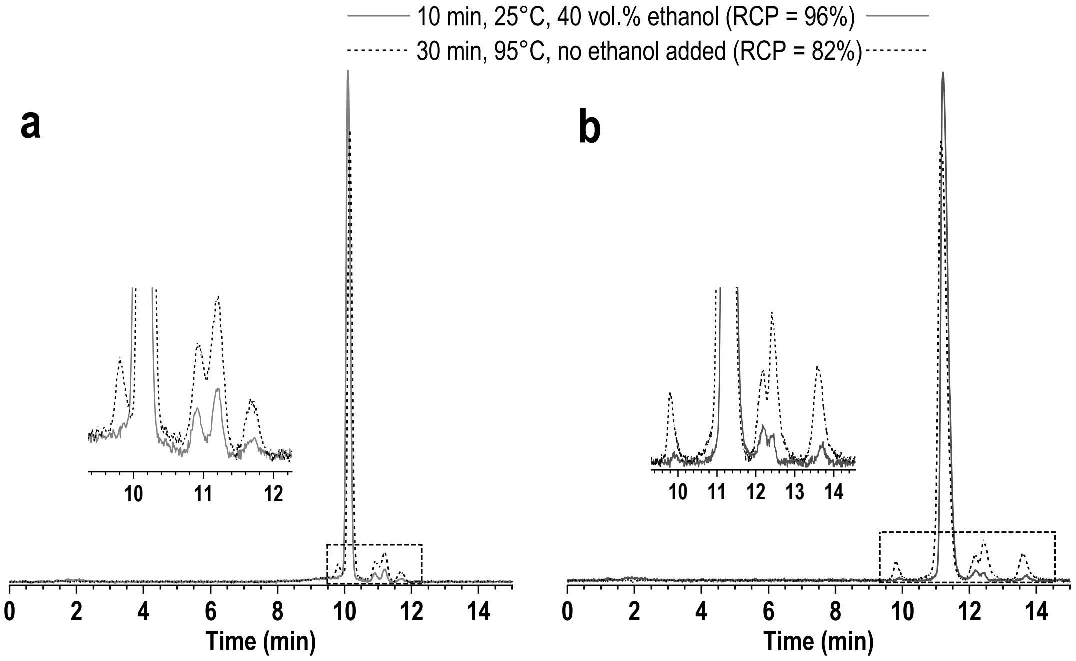

Figure S18. Radio-HPLC chromatograms of the ^{177}Lu-radioconjugates with compounds "13b" (**a**) and "13c" (**b**) obtained at different synthesis conditions: 10 minutes at 25 °C in presence of 40 vol.% of ethanol (solid grey line), 30 minutes at 95°C in aqueous media (no ethanol added). The volume of each reaction mixture was 1 mL, the amount of ligands was 19.2 nmol, the activity of ^{177}Lu was 250 MBq, 0.03 M sodium acetate (pH 4.5).

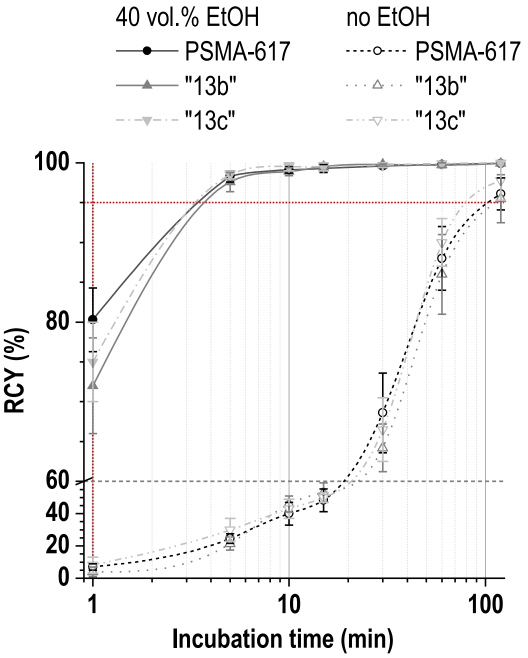

Figure S19. Kinetics of [^{177}Lu]Lu-radioconjugates (PSMA-617, “13b” and “13c”) formation at presence and absence of ethanol at ambient (25 °C) temperature. The volume of the reaction mixture was 1 mL, the amount of each ligand was 19.2 nmol, the activity of ^{177}Lu was 200 MBq, 0.03 M sodium acetate (pH 4.5), $^{nat}LuCl_3$ added, [Lu]:[PSMA] = 1:10. RCY values are presented as mean ± SD, n = 3. The horizontal dashed grey line shows where the Y-scale changes.

References

1. Machulkin, A.E.; Petrov, S.A.; Bodenko, V.; Larkina, M.S.; Plotnikov, E.; Yuldasheva, F.; Tretyakova, M.; Bezverkhniaia, E.; Zyk, N.Y.; Stasyuk, E.; et al. Synthesis and Preclinical Evaluation of Urea-Based Prostate-Specific Membrane Antigen-Targeted Conjugates Labeled with 177 Lu. *ACS Pharmacol. Transl. Sci.* **2024**, *7*, 1457–1473, doi:10.1021/acsptsci.4c00070.
